# Conservation and Divergence in the *Candida* Species Biofilm Matrix Mannan-Glucan Complex Structure, Function, and Genetic Control

**DOI:** 10.1128/mBio.00451-18

**Published:** 2018-04-03

**Authors:** Eddie Dominguez, Robert Zarnowski, Hiram Sanchez, Antonio S. Covelli, William M. Westler, Parastoo Azadi, Jeniel Nett, Aaron P. Mitchell, David R. Andes

**Affiliations:** a Department of Medicine, University of Wisconsin—Madison, Madison, Wisconsin, USA; b Department of Medical Microbiology and Immunology, University of Wisconsin—Madison, Madison, Wisconsin, USA; c National Magnetic Resonance Facility, University of Wisconsin—Madison, Wisconsin, USA; d Complex Carbohydrate Research Center, University of Georgia, Athens, Georgia, USA; e Department of Biological Sciences, Carnegie Mellon University, Pittsburgh, Pennsylvania, USA; Duke University Medical Center

**Keywords:** biofilm, *Candida*, non-*albicans*, antifungal resistance, extracellular matrix

## Abstract

*Candida* biofilms resist the effects of available antifungal therapies. Prior studies with *Candida albicans* biofilms show that an extracellular matrix mannan-glucan complex (MGCx) contributes to antifungal sequestration, leading to drug resistance. Here we implement biochemical, pharmacological, and genetic approaches to explore a similar mechanism of resistance for the three most common clinically encountered non-albicans *Candida* species (NAC). Our findings reveal that each *Candida* species biofilm synthesizes a mannan-glucan complex and that the antifungal-protective function of this complex is conserved. Structural similarities extended primarily to the polysaccharide backbone (α-1,6-mannan and β-1,6-glucan). Surprisingly, biochemical analysis uncovered stark differences in the branching side chains of the MGCx among the species. Consistent with the structural analysis, similarities in the genetic control of MGCx production for each *Candida* species also appeared limited to the synthesis of the polysaccharide backbone. Each species appears to employ a unique subset of modification enzymes for MGCx synthesis, likely accounting for the observed side chain diversity. Our results argue for the conservation of matrix function among *Candida* spp. While biogenesis is preserved at the level of the mannan-glucan complex backbone, divergence emerges for construction of branching side chains. Thus, the MGCx backbone represents an ideal drug target for effective pan-*Candida* species biofilm therapy.

## IMPORTANCE

*Candida* species, the most common fungal pathogens, frequently grow as a biofilm. These adherent communities tolerate extremely high concentrations of antifungal agents, due in large part, to a protective extracellular matrix. The present studies define the structural, functional, and genetic similarities and differences in the biofilm matrix from the four most common *Candida* species. Each species synthesizes an extracellular mannan-glucan complex (MGCx) which contributes to sequestration of antifungal drug, shielding the fungus from this external assault. Synthesis of a common polysaccharide backbone appears conserved. However, subtle structural differences in the branching side chains likely rely upon unique modification enzymes, which are species specific. Our findings identify MGCx backbone synthesis as a potential pan-*Candida* biofilm therapeutic target.

## INTRODUCTION

*Candida* species are among the most common causes of fungal infection worldwide (1). More than a hundred *Candida* species have been identified, but fewer than two dozen have been implicated in human disease. *Candida albicans* is the predominant clinical species; however, other *Candida* species are increasingly encountered (1–5). Four species, Candida albicans, C. tropicalis, C. parapsilosis, and C. glabrata, account for nearly 95% of all infections. While the epidemiology of infection varies among these species, their disease manifestations are similar. Likewise, their general virulence attributes appear mostly conserved (6–17). Among these factors, the ability to live in the biofilm state is arguably responsible for a majority of invasive infections (18–24).

In a biofilm, microbes are protected from antimicrobial agents by an organism-produced extracellular matrix (25–30). Hence, biofilm-related infections are challenging to cure (18, 31–33). The structure, function, and genetic control of this process were recently defined for C. albicans (34–41). Biochemical investigation identified a unique mannan-glucan complex (MGCx) composed of three polysaccharide building blocks, α-1,6-mannan and β-1,6- and β-1,3-glucans (40). These polysaccharides assemble extracellularly to form a complex capable of impeding antifungal delivery through drug sequestration (39, 42). While recent studies have shed light on this process in C. albicans biofilms, only a few studies have begun to explore matrix production and function of other *Candida* spp., and there is a paucity of detailed structure-function knowledge for these emerging pathogens (27, 40, 43–47). Furthermore, the genetic pathways linked to matrix production or function in non-albicans *Candida* species remains unexplored. Elucidation of matrix biogenesis mechanisms in these emergent species thus addresses an intriguing biological question as well as a critical medical need.

Here, we present evidence of a conserved matrix mannan and glucan complex backbone (MGCx) which contributes to profound drug resistance exhibited by the four most prevalent *Candida* species. We show that select matrix synthesis C. albicans orthologs play similar roles across *Candida* species to synthesize a common polysaccharide backbone. However, structural and molecular divergence in matrix assembly is suggested by subtle differences in matrix branching and absence of the involvement of matrix modification enzyme orthologs. Our findings argue that broad-spectrum *Candida* biofilm drug discovery should target the level of MGCx backbone synthesis as opposed to side chain synthesis.

## RESULTS

### Comparison of *Candida* species biofilm growth and architecture.

**FIG 1:**
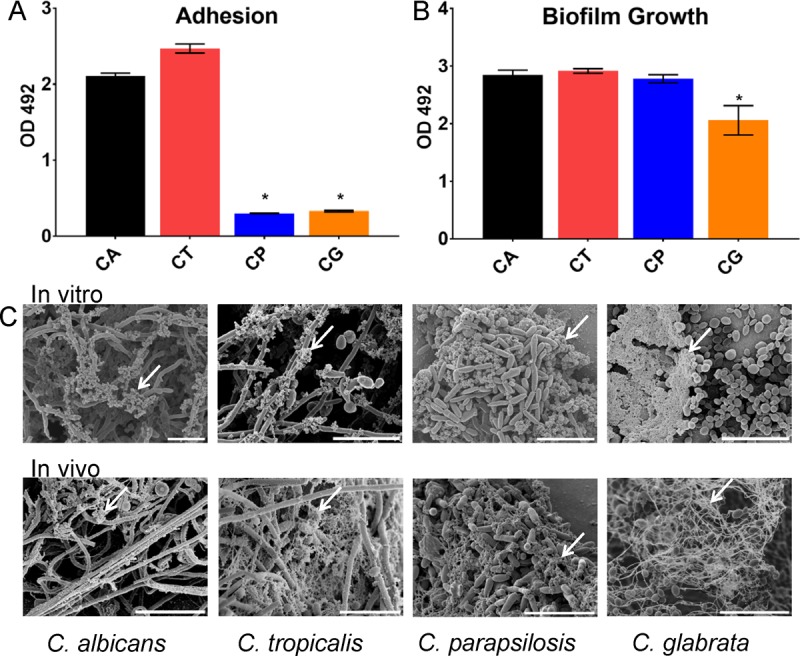
NAC form biofilms with variable characteristics. (A) Biofilm adhesion of reference strains for C. albicans (CA), C. tropicalis (CT), C. parapsilosis (CP), and C. glabrata (CG) was assessed using an XTT assay in a 96-well polystyrene plate after 1 h for adherence. The asterisks indicate statistically significant lower concentrations (*P* < 0.001) for C. parapsilosis and C. glabrata based upon ANOVA using the Holm-Sikak method for pairwise comparison. OD 492, optical density at 492 nm. (B) Mature biofilm formation for each of the four species was quantified in a 96-well format using an XTT endpoint after 24 h of incubation. The asterisk indicates a statistically significant lower concentration (*P* < 0.001) for C. glabrata based upon ANOVA using the Holm-Sikak method for pairwise comparison. (C) Mature biofilm architecture of wild-type biofilms from *in vitro* coverslips and the *in vivo* rat catheter model was assessed visually using SEM imaging after 24 h of incubation. The white arrows indicate extracellular matrix material. Bars, 20 µm.

We determined basic parameters of biofilm development for three non-albicans *Candida* species: C. parapsilosis, C. tropicalis, and the more distantly related C. glabrata. We also included wild-type C. albicans as a standard for comparison. Biofilm formation begins with adherence of cells to the substrate, and we found marked differences among the species in this property (Fig. 1A). Despite similar inocula, the yield of adherent cells was approximately fivefold lower for C. parapsilosis and C. glabrata than that measured for either C. albicans or C. tropicalis. Biofilm maturation over the next 24 h erased these differences in cell number estimates (Fig. 1B), thus indicating that all four species reach similar sessile equilibria under *in vitro* growth conditions. Scanning electron microscopy (SEM) of mature biofilms revealed that each of the species, with the exception of C. glabrata, demonstrated a preponderance of filamentous or elongated cell growth (Fig. 1C). In addition, abundant extracellular matrix material encased the cells within each biofilm (marked by white arrows). We extended our comparison to examine *in vivo* biofilm formed in the lumen of a rat vascular catheter. Basic architecture in this model recapitulated the biofilm characteristics observed *in vitro*: filamentous cells were present in C. albicans, C. parapsilosis, and C. tropicalis biofilms, and extracellular matrix material was abundant. In fact, extracellular matrix appeared more pronounced *in vivo* than *in vitro*, likely due to the contribution of host components (48). These results indicate that mature biofilms of the three non-albicans *Candida* species resemble those of C. albicans in terms of cellular content, matrix accumulation, and for C. parapsilosis and C. tropicalis, presence of filamentous cells.

**FIG 2:**
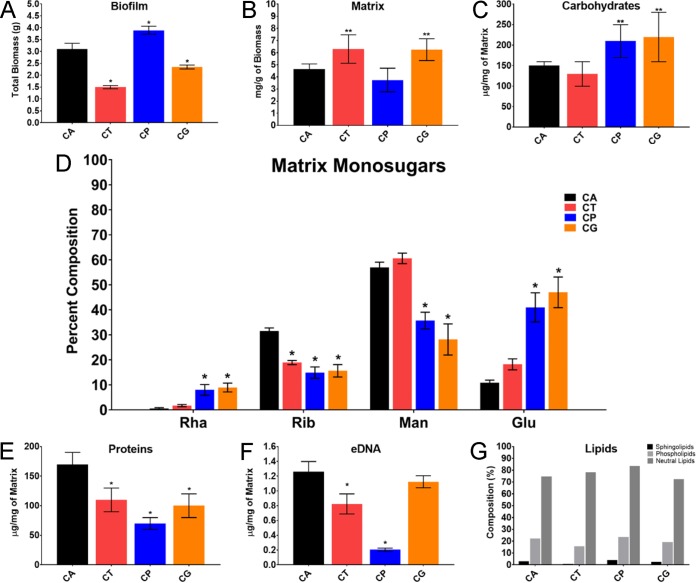
NAC form biofilm matrix of variable quantity and quality. (A) Total biofilm mass was assessed by measurements (dry weight) of *in vitro* biofilms grown in polystyrene roller bottles (three replicates of 20 bottles per species). The four species studied were C. albicans (CA), C. tropicalis (CT), C. parapsilosis (CP), and C. glabrata (CG). The single asterisks indicate statistically significant lower values (*P* < 0.001) for *C. tropicalis* and *C. glabrata* based upon ANOVA using the Holm-Sikak method for pairwise comparison. (B) Biofilm matrix biomass was quantified by the dry weight following matrix separation from biofilm cells. Biofilms were grown in polystyrene roller bottles (three replicates of five bottles per species). Two asterisks indicate statistically lower values for *C. tropicalis* (*P* = 0.003) and *C. glabrata* (*P* = 0.005) between strains based upon ANOVA using the Holm-Sikak method for pairwise comparison. (C) Biofilm matrix total carbohydrate concentration was assessed using the phenol-sulfuric acid assay. The results were normalized by matrix biomass. The single asterisks indicate statistically lower concentrations for *C. parapsilosis* (*P* = 0.009) and *C. glabrata* (*P* = 0.002) based upon ANOVA using the Holm-Sikak method for pairwise comparison. (D) Relative percent monosugar composition (Rha, rhamnose; Rib, ribose; Man, mannose; Glu, glucose) in the biofilm matrix of *C. albicans*, *C. tropicalis*, *C parapsilosis*, and *C. glabrata.* The single asterisks indicate statistically significant differences (*P* < 0.001) between strains based upon ANOVA. (E) Biofilm matrix total protein concentration was assessed using the BCA protein assay kit. The results were normalized by matrix biomass. The single asterisks indicate statistically lower concentrations for *C. tropicalis*, *C. parapsilosis*, and *C. glabrata* (*P* < 0.001) than for *C. albicans* based upon ANOVA using the Holm-Sikak method for pairwise comparison. (F) Biofilm matrix total eDNA. The results were normalized by matrix biomass. The single asterisks indicate statistically lower concentrations for *C. tropicalis*, *C. parapsilosis*, and *C. glabrata* (*P* < 0.001) than for *C. albicans* based upon ANOVA using the Holm-Sikak method for pairwise comparison. (G) Biofilm matrix total lipid concentration was assessed by gas chromatography. The results were normalized by matrix biomass.

To determine the chemical nature of biofilm extracellular matrix material from each species, we used a large-scale, roller bottle apparatus for biofilm production and matrix isolation as described previously (49). Under these conditions, net biofilm biomass was comparable among the species (Fig. 2A), as it was in the small-scale experiments described above. The amounts of biofilm matrix mass (dry weight) in relation to the total biomass were relatively similar across species (Fig. 2B), but the relative abundance of each macromolecular matrix component varied among the species (Fig. 2C to G). Specifically, the protein component was greatest for C. albicans, while the carbohydrate component was greatest for C. parapsilosis and C. glabrata. The extracellular DNA (eDNA) component was comparable among the species (Fig. 2F). These results are consistent with those of a prior investigation (47) and indicate that macromolecular components vary slightly in their relative contributions to overall matrix composition.

### Detection of matrix MGCx in non-albicans *Candida* species.

**FIG 3:**
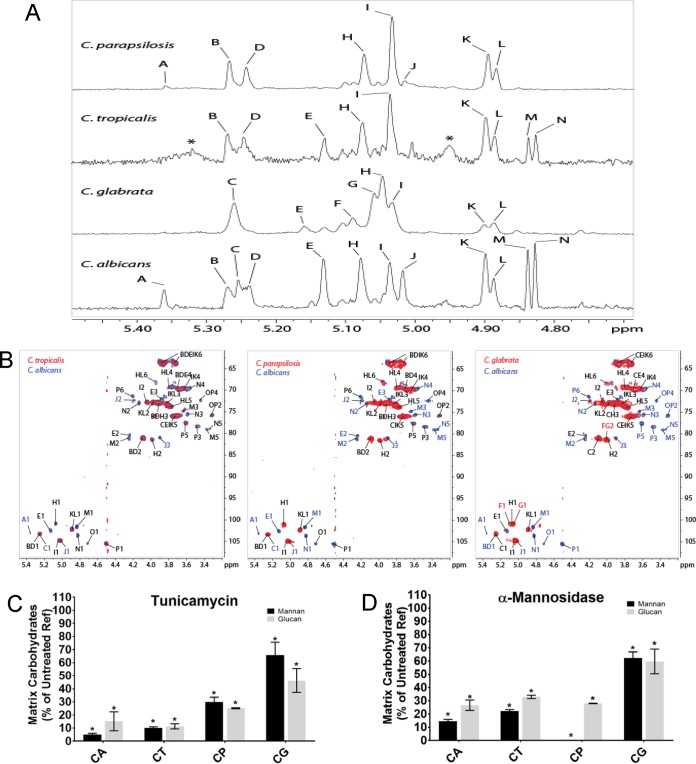
Comparative chromatographic fractionation and NMR analysis of carbohydrates from the *Candida* species biofilm extracellular matrix. (A) Comparison of the 500-MHz ^1^H NMR spectra of purified neutral matrix polysaccharides from *C. albicans*, *C. tropicalis*, *C. parapsilosis*, and *C. glabrata* biofilm matrix. See Table 2. (B) Comparison of *C. albicans* NMR spectra spin systems to each of the NAC species (*C. tropicalis*, *C. parapsilosis*, and *C. glabrata*, respectively) as reflected by heteronuclear single quantum coherence (HSQC) and nuclear Overhauser effect spectroscopy (NOESY) data. (C and D) Carbohydrates in the matrix of wild-type biofilms treated with tunicamycin (C) and α-mannosidase (D) were quantified by gas chromatography. Data are presented as percentages of the reference strain (Ref), with means ± standard errors (SEs) (error bars) shown. All values were significantly lower than the reference value according to ANOVA as indicated by the single asterisks.

The mannan-glucan complex (MGCx) is a signature feature of the C. albicans biofilm (40). The MGCx is comprised of an α-1,6-mannan backbone and β-1,6-glucan (40). Based on our previous report, the MGCx constitute approximately 20% of the total carbohydrate pool in the C. albicans matrix. The MGCx isolated from the non-*albicans Candida* species (NAC) biofilm matrices constituted 13.5%, 34.8%, and 17.0%, in C. tropicalis, C. parapsilosis, and C. glabrata, respectively (Table 1). Further gas chromatography revealed the presence of both mannan and glucan in the matrix of each non-*albicans Candida* biofilm (Fig. 3A). The C. albicans MGCx has a mannan/glucan ratio of 89:11 (40). We found mannan/glucan ratios of 64:25 for C. tropicalis, 83:12 for C. parapsilosis, and 93:7 for C. glabrata (Fig. 3A and Table 1). These findings are consistent with the production of an MGCx by each non-albicans *Candida* species, though the MGCx of each species may have distinct structural features.

**TABLE 1:** Carbohydrate distribution in *Candida* species matrix following neutral sugar purification and fractionation

*Candida* species^a^	Carbohydrate type^b^				
	Bound (%)^c^	Neutral			
		%^c^	MWF^d^	Man/Glu ratio	Content (%)^c^
CT	86.5	13.5	HMWF	72:28	2.6
			HMWF	19:81	4.3
			LMWF	73:27	0.9

CP	65.2	34.8	HMWF	13:87	20.9
			LMWF	10:90	13.9

CG	83.0	17.0	HMWF	95:5	0.4
			HMWF	82:18	1.0
			HMWF	61:39	1.6
			HMWF	52:48	3.4
			HMWF	27:73	0.8
			LMWF	80:20	1.1
			LMWF	50:50	1.7

a CT, C. tropicalis; CP, C. parapsilosis; CG, C. glabrata.

b The carbohydrate type indicates whether carbohydrate is associated either with the uncharged neutral fraction or the charged bound fraction (glycoproteins).

c Values are percentages of the listed fractions in the total matrix carbohydrate pool. Content (%) represents the percent content within the neutral pool for each species.

d MWF is the molecular weight fraction for each isolated polymer within the neutral carbohydrate pool. HMWF and LMWF are the high-molecular-weight-reaction and low-molecular-weight fraction, respectively.

Nuclear magnetic resonance (NMR) analyses performed on total purified NAC MGCx revealed compelling structural similarities as well as striking differences among the neutral matrix polysaccharides of the species (Fig. 3B and C and Table 2; see Table S1 in the supplemental material). Control analysis of C. albicans samples revealed several α- and β-Man spin systems that resembled the high- and low-molecular-weight F2 and F17 glucomannans we reported previously (40). Of particular relevance, we found multiple signals specific for α-1→2-Manα-1→2 residues enclosed within branched regions, such as those found in the MGCx side chains. Several of these spin systems (Fig. 3A and B, peaks B, D, H, I, K, and L) were common to C. albicans, C. parapsilosis, and C. tropicalis. Fewer spin systems were common to C. albicans and C. glabrata (Fig. 3B, peaks C, I, K, and L), as may be expected from their greater phylogenetic distance. We also found peaks characteristic of β1,6-glucan in two-dimensional (2D) NMR analysis (Fig. 3B, peaks P and O). In addition to differences in the presence or absence of specific peaks, differences among the polysaccharide compositions were also evident from the quantitative differences among individual anomeric peaks (Fig. 3B and Table 2).

10.1128/mBio.00451-18.7 TABLE S1  2D HSQC NMR chemical shift assignment of the major spin systems found in C. albicans, C. glabrata, C. tropicalis, and C. parapsilosis. Download TABLE S1, DOCX file, 0.02 MB. Copyright © 2018 Dominguez et al. 2018 Dominguez et al. This content is distributed under the terms of the Creative Commons Attribution 4.0 International license.

**TABLE 2:** Percentages of the different residues found in each sample following 1D 1H NMR

No.	Residue	% of residue in *Candida* species^a^:				% of MGCx^b^	
		CA	CG	CT	CP	F2	F17
i	β-1-2-Manα-1-P					1.8	
ii	α-1-2-Manα-1-P					1.8	0.4
A	α-1-2-Manα-1-3-	3.7			2.1	0.9	1.1
B	α-1-2-Manα-1-2-	4.3		9.5	11.5	5.4	
C	α-1-2-Manα-1-2-	7.2	21.8			7.1	14.1
D	α-1-2-Manα-1-2-	7.4		10.5	12.8	9.3	15.6
E	β-1-2-Manα-1-2-	11.9	5.7	9.1		13	4.1
F	2,6-Manα-1-6- (l)^c^		5.3				
G	2,6-Manα-1-6- (l)		15.1				1.6
H	2,6-Manα-1-6- (b)^d^	15.6	28.2	18.5	18.1	10.8	13.4
I	Manα-1-2-	13.5	14.1	23.7	29.1	10.6	20.9
J	3-Manα-1-2-	7.2			4.4		
K	Manα-1-6-	10.6	6.4	13.5	15.6	16.1	15.8
L	6-Manα-1-6-	6.4	3.5	7.2	6.3	2.4	6.6
M	β-1-2-Manβ-1-2-	5.4		3.7		10.3	3.2
N	Manβ-1-2-	6.7		4.4		10.3	3.2

a CA, C. albicans; CG, C. glabrata; CT, C. tropicalis; CP, C. parapsilosis.  Blank cells indicate that the residue was not found.

b F2 and F17 refer to high- and low-molecular-weight MGCx previously reported in the biofilm matrix of C. albicans (40). Blank cells indicate that the MGCx was not found.

c (l), residues within a linear region, i.e., residues whose neighbors are not branching residues.

d (b), residues within a branched region, i.e., residues whose neighbors are branching residues.

We used a biochemical approach to test for a physical interaction among the matrix polysaccharide components. Specifically, we assessed the glycosyl composition of matrix polysaccharides following a series of neutral sugar purification and fractionation steps. For each species fraction, we identified both mannan and glucan in the high-molecular-weight (HMW) and low-molecular-weight (LMW) fractions. Comigration of both mannan and glucan components strongly suggests the presence of covalent bonds between those two polymers; however, their definite nature is not completely understood. This observation is consistent with coelution of each monosugar as a distinct mannan-glucan complex polysaccharide in the NAC biofilm matrix (Table 1). However, in keeping with analysis described above, the ratios of mannan and glucan varied somewhat across the species, suggesting structural differences in this matrix component among the NAC. In addition, there were several distinct mannan-glucan complexes of different sizes for C. glabrata. These cofractionation results support the model that each non-albicans *Candida* species produces a biofilm matrix MGCx.

We also used pharmacological and enzymatic approaches to test predictions of the MGCx model. Both polysaccharides are required for MGCx assembly in this model; hence, disruption of either mannan or glucan alone would be predicted to decrease matrix accumulation of the other (40). To inhibit matrix mannan production, we utilized tunicamycin, an inhibitor of N-glycosylation, and α-mannosidase, an enzyme that hydrolyzes and degrades mannan (39). Either tunicamycin or α-mannosidase treatment of biofilm reduced the matrix mannan content on average by 28% and 25%, respectively (Fig. 3C and D). Each treatment caused a concomitant decrease in the concentration of glucan that was nearly identical in magnitude (25% and 27%, respectively) (Fig. 3C and D). The finding that matrix glucan accumulation is dependent upon matrix mannan supports the model that polysaccharide interaction leading to an MGCx is conserved across these *Candida* species (39).

### Mannan-glucan matrix function in NAC species.

**FIG 4:**
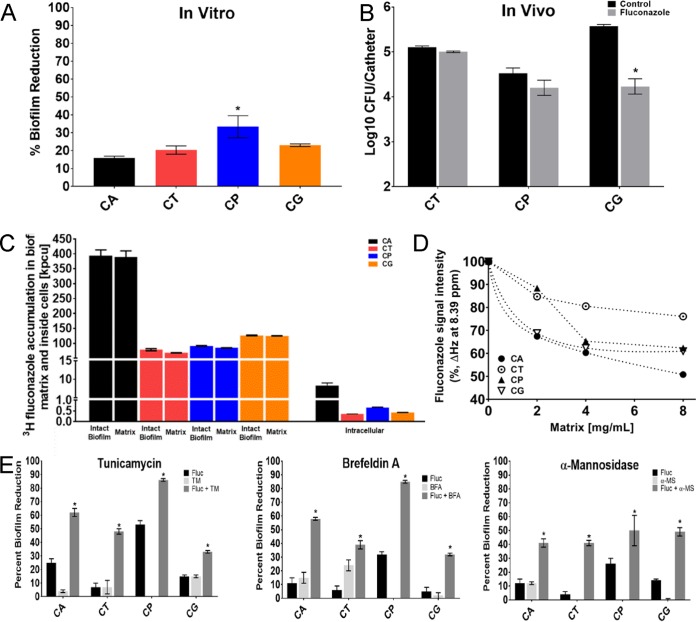
NAC biofilm drug resistance phenotype and mechanism. (A) Biofilm antifungal susceptibility (fluconazole [125 or 1,000 µg/ml] for 48 h) of wild-type C. albicans, C. tropicalis, C. parapsilosis, and C. glabrata was assessed using an XTT assay in a 96-well polystyrene plate assay. The asterisk indicates a statistically significant difference (*P* < 0.001) between strains based upon ANOVA using the Holm-Sikak method for pairwise comparison. (B) Biofilm antifungal susceptibility (fluconazole [250 µg/ml] after 24-h exposure) of wild-type C. albicans, C. tropicalis, C. parapsilosis, and C. glabrata was assessed using viable counts from the rat vascular catheter biofilm model. (C) Fluconazole sequestration and binding to the *Candida* species biofilm extracellular matrix. Sequestration of ^3^H-labeled fluconazole was assessed using *in vitro* intact biofilms as well as the extracellular matrix and intracellular components. (D) Fluconazole binding to the NAC biofilm extracellular matrices. Fluconazole interactions with the tested matrices studied by one-dimensional ^1^H NMR at 600 MHz were determined as decreases in the intensity of chemical shift peaks characteristic of protons present either in the heterocyclic azole rings or the aromatic ring of the drug. Spectra were recorded at the constant fluconazole concentration of 0.653 mM, and matrix concentrations ranged from 0 up to 8 mg/ml. (E) Biofilms were treated with pharmacological inhibitors of mannan or glucan or a mannan hydrolysis enzyme both with and without 1,000 μg/ml fluconazole for all species except C. parapsilosis for which we used 250 μg/ml. Efficacy was assessed in a 96-well plate format for quantification with the XTT assay. The asterisks indicate statistically significant differences (*P* < 0.001) between the combination and either treatment alone based upon ANOVA using the Holm-Sikak method for pairwise comparison. FLUC, fluconazole; TM, tunicamycin; BFA, brefeldin A; α-MS, α-mannosidase.

Prior studies have linked a majority of the C. albicans biofilm resistance phenotype to sequestration of antifungal drugs by the extracellular matrix (28, 34, 36, 37, 39, 50–52). The MGCx appears strongly linked to this drug sequestration phenomenon. Therefore, we examined the drug susceptibility of biofilms both *in vitro* and *in vivo* using a rat vascular catheter model for each of the *Candida* species (53, 54) (Fig. 4A and B). For C. albicans, C. tropicalis, and C. glabrata, the highest soluble concentration of the antifungal fluconazole (1,000 µg/ml, which is more than 1,000 times the planktonic MIC) did not appreciably impact biofilm cell burden in either model, consistent with prior biofilm antifungal testing for these species (55–58). For the strain of C. parapsilosis studied, treatment was more effective compared to the other species. However, the fluconazole concentration needed for effective treatment remained 100 times greater than that associated with planktonic efficacy. These results confirm that biofilms of these species are dramatically less sensitive to fluconazole than planktonic cells. To evaluate drug sequestration as a mechanism underlying the biofilm-associated drug resistance, we tracked radiolabeled fluconazole within the biofilm of each *Candida* species. Nearly all of the antifungal accumulated in the biofilm matrix for each species (Fig. 4C). Interestingly, despite the fact that each species was exposed to a similar concentration of fluconazole, the total sequestered drug concentrations were lower in the NAC biofilms. Because matrix levels are relatively similar among the species (Fig. 2B), we speculate that differences in matrix-drug binding affinity among the species account for differences in drug accumulation.

We further explored the capacity of NAC matrices to interact with fluconazole using one-dimensional ^1^H NMR. In our experiments, interactions of the antifungal drug with the matrix were evident by changes in the intensity of ^1^H peaks upon increasing concentrations of matrix material. Similarly to our previous report (40), the signals of both aromatic and azole protons decreased as a function of increasing concentration of matrices, suggesting that both the aromatic ring and the heterocyclic triazole rings were involved in interactions with matrix components. Interestingly, each NAC matrix had a distinct drug binding dynamics profile (Fig. 4D). The degree of interaction in this assay was greatest for C. albicans, consistent with differences in matrix affinity among the species.

If the MGCx were responsible for functional drug sequestration, then MGCx disruption should increase biofilm drug susceptibility. We tested this prediction through pharmacological and enzymatic treatments. Mannan accumulation was reduced by either tunicamycin or α-mannosidase treatment as shown above. In addition, both mannan and β-1,6-glucan accumulation were reduced by brefeldin A treatment (59). These treatments alone did not influence biofilm cell viability. However, each treatment augmented the activity of fluconazole, resulting in profound killing of biofilm cells (Fig. 4D). These effects were biofilm specific, as these agents did not enhance the activity of fluconazole against planktonic cells (Fig. S1). In sum, these results support the hypothesis that MGCx contributes to sequestration of antifungals to promote biofilm resistance across *Candida* species.

10.1128/mBio.00451-18.1 FIG S1  Planktonic cell growth following pharmacological and enzymatic treatments. Planktonic cells were grown in 96-well round-bottom plates for 24 h and quantified using XTT. For experiments with tunicamycin (TM) (at 1.0 μg/ml) and brefeldin A (BFA) (at 0.6 μg/ml), biofilms or cells were grown for 6 h and then treated for 24 h before quantification. Experiments with α-mannosidase (α-MS) (at 0.78 U/ml) used biofilms or cells first grown for 24 h before 24-h treatment. The means of three technical replicates and SEs are shown. Download FIG S1, TIF file, 2.2 MB. Copyright © 2018 Dominguez et al. 2018 Dominguez et al. This content is distributed under the terms of the Creative Commons Attribution 4.0 International license.

### Genetic determinants of NAC matrix MGCx structure and function.

In order to identify genetic determinants of matrix production across *Candida* species, we used a candidate gene approach. Hypothesizing genetic conservation among species, we identified orthologs of 12 C. albicans genes shown to be involved in production or modification of matrix mannan or β-1,6-glucan (Table 3) (34, 39). We successfully constructed homozygous deletion mutations for the genes in each species with the exceptions of *ALG11* and *VRG4* in C. tropicalis, *BIG1*, *KRE5*, and *VRG4* in C. parapsilosis, and *KRE5* and *VRG4* in C. glabrata. We speculate that these genes may be essential in the respective species. Most of the mutants formed biofilms similar to their reference strains. However, three mutants formed biofilms with reduced cell numbers (Fig. S2), including C. parapsilosis *mnn11*Δ/Δ, *pmr1*Δ/Δ, and *phr1*Δ/Δ mutants and C. glabrata *mnn11*Δ and *big1*Δ mutants.

10.1128/mBio.00451-18.2 FIG S2  Biofilm formation capacity for non-*albicans Candida* mutants in the mannan and glucan pathways. Mature biofilm burden for each of the select deletion mutants was quantified in a 96-well format using an XTT endpoint after 24 h of incubation. Asterisks indicate statistically significant differences (*P* < 0.001) between strains based upon ANOVA. Download FIG S2, TIF file, 4.2 MB. Copyright © 2018 Dominguez et al. 2018 Dominguez et al. This content is distributed under the terms of the Creative Commons Attribution 4.0 International license.

**TABLE 3:** C. tropicalis, C. parapsilosis, and C. glabrata mutant strains used in this study

Species and gene	Systemic name	Genotype	Strain	Description^a^	Homology (%)^b^
C. tropicalis					
*MNN9*	CTRG_02261	Δ/Δ	URZ565	β-1,6-Mannosyltransferase	85.6
*MNN11*	CTRG_04663	Δ/Δ	URZ567	α-1,6-Mannosyltransferase	67.9
*VAN1*	CTRG_05614	Δ/Δ	URZ570	α-1,6-Mannosyltransferase	94.4
*MNN4-4*	CTRG_05766	Δ/Δ	URZ566	Mannosylphosphate transferase	55.2
*PMR1*	CTRG_04916	Δ/Δ	URZ569	Ca^2+^/Mn^2+^ ATPase	88.2
*XOG1*	CTRG_04334	Δ/Δ	URZ571	β-1,3-Glucanase	75.8
*BGL2*	CTRG_00169	Δ/Δ	URZ562	β-1,3-Glucosyltransferase	61.5
*PHR1*	CTRG_03942	Δ/Δ	URZ568	β-1,3-Glucosyltransferase	75.2
*BIG1*	CTRG_04070	Δ/Δ	URZ563	β-1,6-Glucan synthesis	70.3
*KRE5*	CTRG_02572	Δ/Δ	URZ564	β-1,6-Glucan synthesis	65.7
C. parapsilosis					
*ALG11*	CPAR2_601300	Δ/Δ	EGD136	α-1,2-Mannosyltransferase	58.4
*MNN9*	CPAR2_806810	Δ/Δ	EGD194	α-1,6-Mannosyltransferase	78.2
*MNN11*	CPAR2_106380	Δ/Δ	EGD144	α-1,6-Mannosyltransferase	55.9
*VAN1*	CPAR2_807920	Δ/Δ	EGD184	α-1,6-Mannosyltransferase	73.7
*MNN4-4*	CPAR2_106570	Δ/Δ	EGD149	Mannosylphosphate transferase	36.0
*PMR1*	CPAR2_31360	Δ/Δ	EGD141	Ca^2+^/Mn^2+^ ATPase	82.7
*XOG1*	CPAR2_106000	Δ/Δ	EGD150	β-1,3-Glucanase	64.2
*BGL2*	CPAR2_401600	Δ/Δ	EGD147	β-1,3-Glucosyltransferase	72.7
*PHR1*	CPAR2_302140	Δ/Δ	EGD188	β-1,3-Glucosyltransferase	59.7
C. glabrata					
*ALG11*	CAGL0D01122g	Δ	EGD125	α-1,2-Mannosyltransferase	29.5
*MNN9*	CAGL0L12804g	Δ	EGD128	α-1,6-Mannosyltransferase	52.4
*MNN11*	CAGL0G07491g	Δ	EGD124	α-1,6-Mannosyltransferase	25.7
*VAN1*	CAGL0B02321g	Δ	EGD137	α-1,6-Mannosyltransferase	49.4
*MNN4-4*	CAGL0H01793g	Δ	EGD121	Mannosylphosphate transferase	26.4
*PMR1*	CAGL0J01870g	Δ	EGD143	Ca^2+^/Mn^2+^ ATPase	60.0
*XOG1*	CAGL0G09515g	Δ	EGD134	β-1,3-Glucanase	52.2
*BGL2*	CAGL0G00220g	Δ	EGD127	β-1,3-Glucosyltransferase	65.3
*PHR1* (*GAS2*)	CAGL0M13849g	Δ	EGD131	β-1,3-Glucosyltransferase	57.1
*BIG1*	CAGL0L11528g	Δ	EGD129	β-1,6-Glucan synthesis	23.2

a Based on *Candida* or *Saccharomyces* Genome Database.

b Protein sequence alignment to *Candida albicans*.

**FIG 5:**
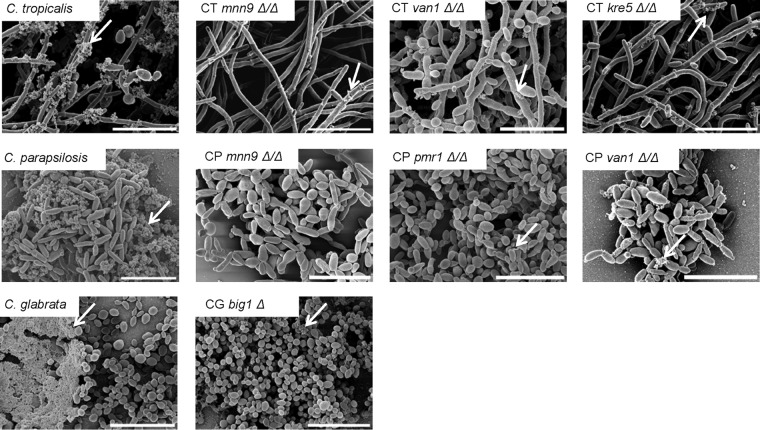
Genetic control of NAC biofilm extracellular matrix production. Mature biofilm architecture from *in vitro* coverslips was assessed visually using SEM imaging after 24 h of incubation. The white arrows indicate extracellular matrix material. Bars, 20 µm.

We examined each of the mutant biofilms by electron microscopy to visualize biofilm architecture and matrix deposition. Biofilm architecture seemed largely unaffected by the mutations, but there was a striking reduction of visible extracellular matrix in seven of the mutant biofilm strains (Fig. 5). This suggests that each of these genes is required for biofilm matrix production in the respective NAC species.

**FIG 6:**
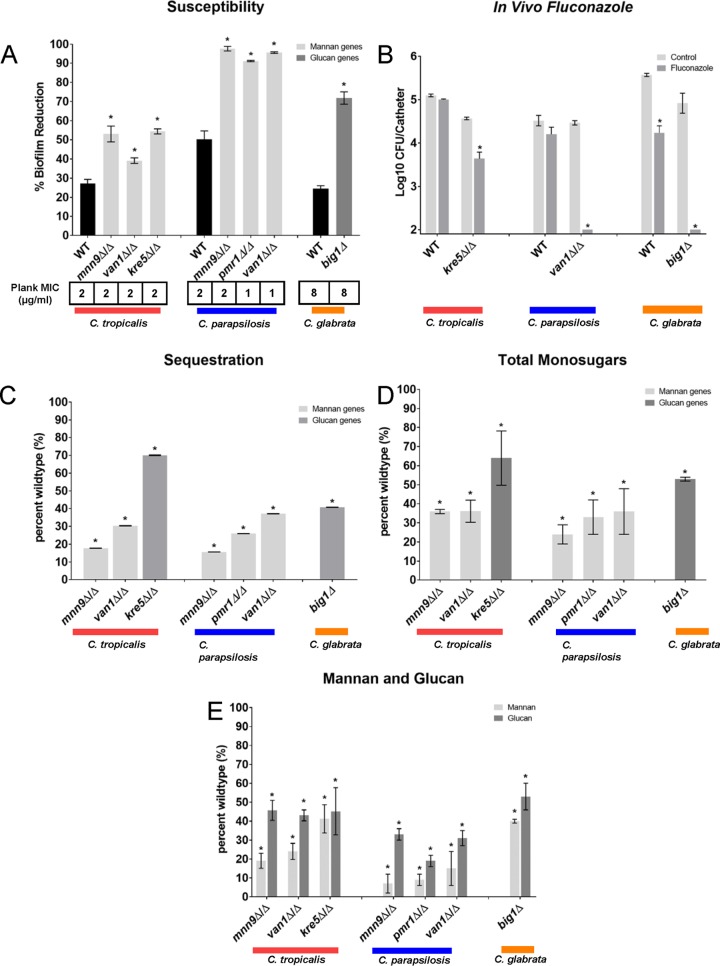
Genetic control of NAC biofilm drug resistance and extracellular matrix production. (A) The percentage of reduction in biofilm formation following 48-h treatment with fluconazole compared with untreated biofilms, as quantified using the 96-well XTT assay. The null mutant (Δ/Δ) is shown for each gene of interest. The graph shows data from three assay replicates of a representative example of three biological replicates. Asterisks indicate statistically significant difference (*P* < 0.001) between the reference (wild type [WT]) and mutant based upon ANOVA using the Holm-Sidak method for pairwise comparison. The MIC of fluconazole for planktonic cells of the Δ/Δ strains is shown below the bar graph by the Plank MIC values. (B) The NAC species reference strains, *kre5*Δ/Δ, *van1*Δ/Δ, and *big1*Δ/Δ mutants, were tested *in vivo* using a rat central venous catheter model, with the effects of fluconazole or saline treatment compared with the reference strains for C. tropicalis, C. parapsilosis, and C. glabrata, respectively. Biofilms were quantified using viable-cell counts following treatment. The values are means ± standard deviations (error bars) from three replicates. The asterisks indicate that the CFU values were significantly different from the CFU for the reference strain (*P* < 0.001) based upon ANOVA using the Holm-Sikak method for pairwise comparison. (C) Intact biofilms grown from the wild-type and mutant strains were exposed to [^3^H]fluconazole, washed, and harvested. Scintillation counting was performed in triplicate to determine the fluconazole content in the intact biofilms and the isolated matrix. Standard deviations are shown. (D) Mature *in vitro* biofilms from the wild-type strain and null mutants were assayed for matrix carbohydrate concentration using the phenol sulfuric acid method. The value for each mutant is presented as a percentage of the value for the wild-type strain. The graph shows data from three biological replicates and three assay replicates. The asterisks indicate that glucan measurements were significantly different (*P* < 0.0001) from the wild-type measurement based on ANOVA. (E) Mature *in vitro* biofilms from the wild-type strain and null mutants were assayed for matrix glucan and mannan concentrations by gas chromatography. The value for each mutant is presented as a percentage of the value for the wild-type strain. The graph shows data from three biological replicates and three assay replicates. The asterisks indicate that glucan measurements were significantly different (*P* < 0.0001) from the value for the wild-type strain based upon ANOVA.

We next examined the influence of the genetic disruptions on the biofilm resistance phenotype *in vitro* (Fig. 5 and Fig. S3 and S4). Surprisingly, despite the importance of each of these genes for biofilm resistance in C. albicans, only a small subset impacted susceptibility in the NAC biofilms. Specifically, we found a total of 7 of 29 deletion mutants exhibiting enhanced biofilm drug susceptibility (Fig. 6A). For C. tropicalis, fluconazole-susceptible strains included mannan synthesis mutants, i.e., *mnn9*Δ/Δ and *van1*Δ/Δ mutants, and the β-1,6-glucan synthesis mutant, the *kre5Δ/Δ* mutant. Among the C. parapsilosis mutants screened, we identified three mannan synthesis mutants, *mnn9*Δ/Δ, *van1Δ/Δ*, and *pmr1Δ/Δ* mutants. Interestingly, two of the C. parapsilosis orthologs were also relevant for C. tropicalis (*mnn9Δ/Δ* and *van1Δ/Δ*). Only a single mutant from C. glabrata exhibited a biofilm susceptibility phenotype: a putative enzyme for β-1,6-glucan synthesis (*big1Δ*). The susceptibility to fluconazole appeared specific to the biofilm state, as planktonic MICs for all mutants were unchanged (Fig. 6A). The biofilm susceptibility phenotype was reversed for all strains in which we reintroduced a wild-type copy of the deleted gene (Fig. S5).

10.1128/mBio.00451-18.3 FIG S3  NAC biofilm drug susceptibility in mutants without an enhanced susceptibility phenotype. (A to C) Percentage of reduction in biofilm formation following 48-h treatment with fluconazole compared with untreated biofilms, as quantified using the 96-well XTT assay. The null mutant (Δ/Δ) is shown for each gene of interest. The figure shows data from three assay replicates of a representative example of three biological replicates. Asterisks indicate statistically significant differences (*P* < 0.001) between reference and mutant based upon ANOVA using the Holm-Sikak method for pairwise comparison. Download FIG S3, TIF file, 2.7 MB. Copyright © 2018 Dominguez et al. 2018 Dominguez et al. This content is distributed under the terms of the Creative Commons Attribution 4.0 International license.

10.1128/mBio.00451-18.4 FIG S4  Polyene and echinocandin biofilm activity against fluconazole-susceptible NAC mutants. After growth for 6 h, biofilms were treated with either 0.5 μg/ml amphotericin B or micafungin for 48 h. Biofilms were quantified using the 96-well XTT assay, and reduction was determined by comparing treated and untreated biofilms. All mutants were significantly more susceptible to treatment than the reference strain (*, *P* < 0.001; **, *P* < 0.05). The means of three technical replicates and SEs are shown. Download FIG S4, TIF file, 2 MB. Copyright © 2018 Dominguez et al. 2018 Dominguez et al. This content is distributed under the terms of the Creative Commons Attribution 4.0 International license.

10.1128/mBio.00451-18.5 FIG S5  Complemented *C. tropical* and C. glabrata glucan and mannan synthesis mutant biofilms are susceptible to fluconazole. After growth for 6 h, biofilms were treated with 1,000 μg/ml fluconazole for 48 h. Biofilms were quantified using the 96-well XTT assay, and reduction was determined by comparing treated and untreated biofilms. All mutants were significantly more susceptible to treatment than the reference strain (*P* < 0.001). Each of the complemented strains exhibited resistance similar to that of the reference strains. The means of three technical replicates and SEs are shown. Download FIG S5, TIF file, 1.8 MB. Copyright © 2018 Dominguez et al. 2018 Dominguez et al. This content is distributed under the terms of the Creative Commons Attribution 4.0 International license.

For three C. parapsilosis mutants (*mnn9Δ/Δ*, *pmr1Δ/Δ*, and *van1*Δ/Δ mutants), complementation was not successful; consequently, we examined multiple independent deletion mutant strains to verify the enhanced antifungal susceptibility phenotype (Fig. S6). Antifungal biofilm susceptibility was similarly assessed for two additional drugs, amphotericin B and micafungin (Fig. S4). Enhanced susceptibility was observed for the majority of mutants, consistent with a pan-antifungal mechanism. These results show that inferred defects in mannan or β-1,6-glucan production can cause increased biofilm drug susceptibility in NAC species.

10.1128/mBio.00451-18.6 FIG S6  Multiple independent transformants of noncomplemented mutant strains are susceptible to fluconazole. After growth for 6 h, biofilms were treated with 1,000 μg/ml fluconazole for 48 h. Biofilms were quantified using the 96-well XTT assay, and reduction was determined by comparing treated and untreated biofilms. Two or three independent transformants were tested for each homozygous deletion mutant. All mutants were significantly more susceptible to treatment than the reference strain (*P* < 0.001). A different biological replicate is presented here than in Fig. 5A. The means of three technical replicates and SEs are shown. Download FIG S6, TIF file, 1.9 MB. Copyright © 2018 Dominguez et al. 2018 Dominguez et al. This content is distributed under the terms of the Creative Commons Attribution 4.0 International license.

We explored the clinical relevance of these findings using the rat catheter *in vivo* biofilm model. Enhanced fluconazole efficacy was observed for representative mannan or β-1,6-glucan synthesis mutants from each species (Fig. 6B). These results support the hypothesis that the increased biofilm drug susceptibility that results from impaired mannan or β-1,6-glucan synthesis is relevant in a model infection environment.

To determine directly whether matrix mannan and glucan are required for drug sequestration in the NAC biofilm mutants, we conducted [^3^H]fluconazole binding assays. Compared to the respective wild-type strain, each of the mannan and glucan mutant strains had decreased matrix sequestration of radiolabeled drug (Fig. 6C). These findings strengthen the conclusion from our pharmacological and enzymatic matrix disruption treatments that mannan and glucan contribute to matrix drug sequestration. However, variability in the degree of sequestration relative to the change in susceptibility suggests the potential of additional mechanisms as previously described (28).

To identify matrix changes associated with increased biofilm susceptibility, we harvested matrix from biofilms of each mutant and assayed for total carbohydrate and specifically for mannan and glucan (Fig. 6D and 5E). We found that all seven mutants had significantly lower levels of the corresponding polysaccharide than the reference strain with remaining amounts less than 50% of the level in the wild type on average (Fig. 6E). Furthermore, genetic disruption of either a mannan or glucan synthase was associated with a lower content of both mannan and glucan. This observation is consistent with the model that the MGCx accounts for the drug resistance phenotype of the NAC biofilms. Unfortunately, matrix quantities did not allow for complementary NMR analysis. Because the synthesis pathways for the carbohydrates are distinct, inhibition of one pathway would not be expected to lead to a biochemical reduction in the nonimpacted pathway components. Our findings suggest that there is an extracellular physical interaction among the matrix components that is conserved among *Candida* species.

## DISCUSSION

We theorized that the MGCx, recently found in C. albicans, is conserved and promotes the drug resistance phenotype across *Candida* species. In order to address this question, in this study, we first utilized large-scale biofilm growth and matrix composition analysis. We find that each of the major matrix polysaccharide constituents is required for assembly and function of matrix across *Candida* species. At the structural level, several complementary assays corroborate the presence of an MGCx in diverse *Candida* species. Specifically, we identified an α-1,6-mannan backbone with α-1,2 branches and β-1,6-glucan in the matrix. Analysis of the glycosyl composition of matrix after fractionation also revealed coelution of mannan and glucan components. Additionally, both pharmacological and genetic studies revealed codependence of accumulation of matrix mannan and glucan. Phenotypic studies documented the central role of this polysaccharide complex in matrix sequestration and biofilm-specific drug resistance under both *in vitro* and *in vivo* conditions.

However, the results of refined NMR analysis of the complex and phenotypic and genotypic assays suggested meaningful differences in the MGCx among the *Candida* species. Specifically, the relative ratios of the mannan and glucan components varied among the four species. Additionally, while NMR analysis revealed similar mannan and glucan backbone features, there were differences in branching pattern and length. The C. parapsilosis matrix mostly resembled low-molecular-weight glucomannans of C. albicans. The abundance of α-1→6-linked Man residues along with a substantially increased content of terminal Manα-1→2 residues indicated the presence of a similar α-1→6-linked Man backbone structure but with shorter side chains consisting of a blend of α-1→2- and α-1→3-linked Man residues. The lack of β-Man for C. parapsilosis matrix was unique, as β-Man was observed for the other species. The matrix of C. tropicalis exhibited characteristics similar to those of C. albicans and C. parapsilosis matrix. Like the latter, this pool contained amounts of α-1→2-Manα-1→2- and 1→6-linked Man as well as of β-Man residues that were comparable to those measured in C. albicans. On the other hand, C. tropicalis matrix neutral carbohydrates did not contain any 3-linked Man residues, whereas there was a larger terminal Manα-1→2 residue content, along with more 1→6-linked Man. This pattern suggests more branching but relatively shorter side chains. On the basis of measured distribution of 2,6-Manα-1→6 and α-1→2-Manα-1→2 residues, it appeared that the overall branching pattern is bit less diverse than that observed in C. albicans matrix. The analysis of C. glabrata matrix neutral carbohydrates revealed the most distinct highly branched structure, which also contained well-defined linear regions without any branching Man residues present. This observation suggested an uneven and infrequent distribution of side chains in this carbohydrate pool as reflected by the presence of only one type of α-1→2-Manα-1→2 signal, whereas the amount of the terminal Manα-1→2 residue indicated a side chain length distribution type resembling the distribution determined in C. albicans. Unlike the latter, C. glabrata matrix neutral carbohydrates did not contain 3-linked Man residues, while the content of β-Man was significantly reduced but still detected. Overall, the major Man residues were similar among all four tested *Candida* species. However, the relative proportion of the residues varied significantly, likely due to differences in the degree of branching, the length of side chains, and the presence of unique residues such as β-Man or 3-linked Man. We speculate that these structural differences in the MGCx and potentially other noncarbohydrate constituents determine the unique sequestration properties of the NAC.

Our genetic studies reveal both similarities and differences among the species with regard to the genetic pathways responsible for production of matrix. It was initially surprising that the only orthologous mutations in the NAC that impacted the antifungal resistance phenotype were those linked to component synthesis alone. Conversely, the mutations with putative glucan and mannan modification function based upon the C. albicans MGCx structure had no apparent impact on NAC matrix production. A plausible explanation for this observation is divergence in the genetic pathways responsible for the differences in the impact of C. albicans orthologous mutations in the NAC species. Researchers have often assumed that if a yeast species is related to another yeast species (especially within the same genus), the underlying molecular and cellular mechanisms must also be closely related. However, even within a *Candida* clade, the genetic relatedness between any two NAC species is often larger than the genetic distance between humans and reptiles (60). There is ample precedent for genetic rewiring among the *Candida* species, including for biofilm formation pathways (13, 15, 16, 61–63). While sequence identity is not a great predictor of function, the amino acid similarity across species for these C. albicans matrix resistance genes was only modest.

Another potential explanation for the lack of genetic conservation is the distinct differences in MGCx branching structure. We favor this model due to the biochemical differences that support a model of altered matrix branch chain structure across species. The latter model suggests that enzymes needed for production of the matrix backbone may be useful for pan-*Candida* biofilm development of useful therapies, while targeting modification enzymes would be predicted to be less effective across species. In future work, careful characterization of the remaining genetic components of the NAC biofilm synthesis pathway will help to elucidate additional therapeutic targets.

## MATERIALS AND METHODS

### Ethics statement.

All animal procedures were approved by the Institutional Animal Care and Use Committee at the University of Wisconsin according to the guidelines of the Animal Welfare Act, the *Guide for the Care and Use of Laboratory Animals* (64), and Public Health Service policy. The approved animal protocol number is DA0031.

### Media.

Strains were stored in 15% (vol/vol) glycerol stock at −80°C and maintained on yeast extract-peptone-dextrose (YPD) agar. Prior to biofilm experiments, all *Candida* strains were grown at 30°C in YPD, and biofilms were grown in RPMI 1640 buffered with morpholinepropanesulfonic acid (RPMI-MOPS).

### Strains and strain construction.

Strains used for this study are listed in Table 3, and the genotypes of strains constructed in the present studies are shown in Table S2 in the supplemental material. The parent strains CAY3764, CPL2H1, and HTL were used to create homozygous deletion strains using fusion PCR disruption cassettes as previously described for C. tropicalis, C. parapsilosis, and C. glabrata, respectively (6, 65–67). Correct integration was confirmed by PCR. At least two independent mutants were created for each gene of interest. The primers used for strain construction and confirmation are listed in Table S3.

10.1128/mBio.00451-18.8 TABLE S2  C. tropicalis, C. parapsilosis, and C. glabrata mutant strains developed in this study. A list of genes under study, strain names, and strain genotypes for the strains constructed and utilized in these studies is shown. Download TABLE S2, DOCX file, 0.02 MB. Copyright © 2018 Dominguez et al. 2018 Dominguez et al. This content is distributed under the terms of the Creative Commons Attribution 4.0 International license.

10.1128/mBio.00451-18.9 TABLE S3  Primers used for mutant strain creation and confirmation. The primers used for genetic modification of strains and the primers used to confirm correct modifications are listed. Download TABLE S3, DOCX file, 0.02 MB. Copyright © 2018 Dominguez et al. 2018 Dominguez et al. This content is distributed under the terms of the Creative Commons Attribution 4.0 International license.

Construction of C. tropicalis mutant strains was conducted using the background strain of CAY3764, which is auxotrophic for *LEU2* and *HIS1* (67). The primers used for strain construction and confirmation are listed in Table S3. For C. parapsilosis strain construction, we used the background strain of CPL2H1, which is auxotrophic for *LEU2* and *HIS1*, as described by Holland et al. (6). The first allele was deleted by replacing one allele with *HIS1* from C. dubliniensis, and the second with *LEU2* from C. maltosa. C. glabrata transformations were conducted using auxotrophic background strain HTL (*HIS3*, *LEU2*, *TRP1*) (63). Candidate genes were deleted by replacing the allele with the gene conferring resistance to the antibiotic nourseothricin. All mutant strains were confirmed by PCR using primers inside nourseothricin and a primer outside the integration sites at both the 5′ and 3′ ends of the gene.

Complementation of C. tropicalis and C. parapsilosis mutant strains with a single wild-type gene copy used selection for nourseothricin resistance, whereas complementation of C. glabrata mutant strains used selection for hygromycin B resistance. A single copy of the gene was reintroduced to its endogenous location within the genome. Briefly, each open reading frame (ORF) (plus 1 kb upstream and downstream) was amplified by PCR, and using the PCR fusion method, a cassette was created with either hygromycin B or nourseothricin at the tail end of the PCR product. This fusion PCR product was then transformed into their respective species using the same method as previously described for mutant construction. Colony PCR was used to verify all genotypes; the primers are listed in Table S3.

### *In vitro* biofilm models.

Biofilms were grown in one of four models: 96-well or 6-well polystyrene plate, polystyrene roller bottle, or glass coverslip. Ninety-six-well flat-bottom polystyrene plates were used to assess biofilm adherence, maturation, and treatment effect as previously described (68, 69). The *Candida* species inocula (10^6^ cells/ml) were prepared by growth in YPD with uridine overnight at 30°C, followed by dilution in RPMI-MOPS based on hemocytometer counts. The six-well plate assay was used to assess matrix composition. For this assay, 1 ml of culture was inoculated in each well. After a 60-min adherence period at 30°C, the nonadherent inoculum was removed and 1 ml of fresh medium (RPMI-MOPS) was applied to each well. The plates were incubated at 37°C for 48 h on an orbital shaker set at 50 rpm. The medium was removed, and fresh medium was added midway through the incubation period. The coverslip assay was used for *in vitro* biofilm imaging. Briefly, *in vitro* biofilms were grown on sterile coverslips (Thermanox) in sterile 12-well plates that had previously been coated with 10 µl of human NaEDTA plasma each and allowed to dry at 30°C. Forty microliters of yeast in RPMI was counted and diluted as in the biofilm models described above and added to each coverslip for 60 min at 30°C. The initial inoculum was then removed, 1 ml of RPMI-MOPS plus 5% NaEDTA human plasma was added to each well, and the plates were incubated for 20 h at 37°C and 50 rpm on an orbital shaker for an additional 24 h. A rolling bottle system was used to generate matrix for analyses (49). Briefly, aliquots of C. albicans grown in RPMI-MOPS were used to inoculate a polystyrene roller. Bottles were placed on a roller apparatus (Wheaton Science Products, Millville, NJ), rolling at the rate of 20 rpm at 37°C. After 24 h, the biofilm culture medium was replaced with fresh medium, and the bottles were incubated for another 24 h. At least three biological replicates were performed for each assay.

### *In vitro* biofilm and planktonic antifungal susceptibility testing.

A tetrazolium salt XTT [2,3-bis-(2-methoxy-4-nitro-5-sulfophenyl)-2H-tetrazolium-5-carboxanilide inner salt] reduction assay was used to measure *in vitro* biofilm drug susceptibility (70, 71). Biofilms were formed in the wells of 96-well microtiter plates as described above. After a 6-h biofilm formation period, the biofilms were washed with phosphate-buffered saline (PBS) twice to remove nonadherent cells. Fresh RPMI-MOPS and drug dilutions were added, followed by additional periods of incubation (48 h). The antifungal studies included fluconazole at 4 to 1,000 mg/ml, amphotericin B at 125 to 0.5 µg/ml, and micafungin at 125 to 0.5 µg/ml. For experiments with tunicamycin (1.0 µg/ml) and brefeldin A (0.6 µg/ml), biofilms were treated alone or in combination with fluconazole after an initial 6-h growth phase. Biofilms treated with α-mannosidase (0.78 U/ml; jack bean; Sigma) were grown for 24 h before a 24-h dose either alone or in combination with fluconazole. Drugs were reapplied after 24 h, and the plates were incubated for an additional 24 h. Following treatment with 90 µl XTT (0.75 mg/ml) and 10 µl phenazine methosulfate (3.20 mg/ml) for 30 min, absorbance at 492 nm was measured using an automated plate reader. The percent reduction in biofilm growth was calculated using the reduction in absorbance compared to that of controls with no antifungal treatment. Assays were performed in triplicate, and significant differences were measured by analysis of variance (ANOVA) with pairwise comparisons using the Holm-Sidak method. The CLSI M27 A3 broth microdilution susceptibility method was used to examine the activities of fluconazole against planktonic *Candida*. sp. (72). Endpoints were assessed after 24 h by visible turbidity in triplicate assays.

### Biofilm SEM.

*In vitro* biofilms from sterile coverslips were grown as described above. Following a 24-h incubation period, the medium was replaced with 1 ml of fixative (4% formaldehyde, 1% glutaraldehyde in PBS), and the coverslips were incubated at 4°C for 24 h. The coverslips were then washed with PBS and treated with 1% osmium tetroxide for 30 min at ambient temperature. After a series of alcohol washes (30 to 100%), final desiccation was performed by critical point drying. Coverslips were mounted, palladium-gold coated, and imaged in a scanning electron microscope (SEM) (LEO 1530) at 3 kV. The images were assembled using Adobe Photoshop 7.0.1.

### Matrix isolation from roller bottle and six-well biofilms.

A rolling bottle system was used to generate matrix for composition analyses (49). After incubation for 48 h, the medium was removed, and the *Candida* biofilms were dislodged with a spatula and gently sonicated to avoid cell wall disruption (sonication with a 6-mm microtip at 20 kHz with an amplitude of 30% for 8 min). The aggregate biofilm was then centrifuged to separate fungal cells and matrix. The supernatant-containing matrix was then collected and lyophilized. The sample was resuspended in water and dialyzed (molecular size cutoff of 3.5 kDa) for 5 days and again lyophilized, yielding the “crude” biofilm matrix. Overall, a total of 400 bottles of the matrix corresponding to the biofilm area of 59.5 m^2^ were collected for analysis.

Matrix was similarly collected from six-well plates as described previously (34). Following incubation, biofilms were harvested by removing and discarding the medium, washing each well with 1 ml of double-distilled water (ddH_2_O), and then removing the biofilms using a spatula. Biofilms were collected in 1 ml of ddH_2_O per well and then sonicated for 20 min. The soluble matrix was then separated from the cells by centrifuging the samples at 2,880 × *g* for 20 min at 4°C.

### Biofilm matrix analysis. (i) Dry weight.

Following biofilm matrix collection described above, matrix was lyophilized, and weighed to obtain total biomass for each biofilm.

### (ii) Carbohydrate analysis.

The carbohydrate concentration of crude matrix was determined colorimetrically (492 nm) using the phenol-sulfuric acid method (73). Structural analysis was performed after a series of purification and fractionation steps, including size exclusion chromatography.

### (iii) Protein analysis.

The protein concentration of the crude matrix sample was assessed colorimetrically at 562 nm using the BCA protein assay kit (Pierce Biotechnology, Rockford, IL) (74).

### (iv) Nucleic acid analysis.

Nucleic acid concentrations were measured spectrophotometrically (260 nm) (75).

### (v) Monosaccharide analysis.

Sugars were detected and quantified by gas-liquid chromatography with a flame ionization detector (GLC-FID) on a Shimadzu GC-2010 system after conversion to alditol acetate derivatives as previously described (76). A 50% cyanopropylmethyl–50% phenylmethyl polysiloxane column was used (Restek) under the GLC conditions previously described (77). Data for each monosaccharide were calculated and presented as a percentage of the total detected sugars.

### (vi) Lipid analysis.

Lipids were extracted from the desalted lyophilized matrix powder with a mixture of CHCl_3_ and methanol (MeOH) (2:1, by volume) as described elsewhere (78). Methylation of fatty acids was performed using 0.5 ml of 14% boron trifluoride (BF3) in MeOH, and methyl esters were recovered with hexane. Fatty acid methyl esters were analyzed by gas chromatography using a Hewlett-Packard 5890 instrument (Hewlett Packard, Palo Alto, CA).

### (vii) eDNA analysis.

Extracellular DNA (eDNA) was isolated from non-*albicans Candida* species (NAC) matrices using the MasterPure yeast DNA purification kit (Epicentre Biotechnologies, Madison, WI). Nucleic acid concentrations were measured spectrophotometrically with a NanoDrop 1000 spectrophotometer (Thermo Fisher Scientific, San Jose, CA). The average measured ratio of absorbance at 260/280 nm was about ~1.8, which indicated that the DNA was pure and free of contaminants.

### NMR of neutral carbohydrates.

After isolation, matrix samples were resuspended in 1 ml of 20 mM bis-Tris HCl (pH 6.5) loading buffer and fractionated on a HiPrep 26/10 desalting column prepacked with Sephadex G-25 Fine (GE Healthcare Life Sciences, Uppsala, Sweden). Column-dialyzed fractions were then separated on an anion exchanger HiPrep 16/10 DEAE FF column (GE Healthcare Life Sciences) equilibrated with 20 mM bis-Tris HCl (pH 6.5). Elution was performed in a 20 mM bis-Tris HCl (pH 6.4)–0.5 M NaCl buffer system at a flow rate of 1 ml/min in a linear gradient of salt from 0 to 100% in 20 column volumes. Neutral free carbohydrates were detected in flowthrough fractions, which were then pooled, lyophilized, resuspended in 2 ml of 150 mM NH_4_HCO_3_, and applied to gel filtration on a Superdex 200 10/300 GL column (GE Healthcare Life Sciences). Matrix components were eluted at a flow rate of 0.5 ml/min, and 1-ml fractions were collected. All chromatographic separation steps were performed at room temperature on the high-performance liquid chromatography Äkta-Purifier 10 system (GE Healthcare Life Sciences). All buffers used were filtered through 0.2-μm nylon membrane filters (Nalgene, Rochester, NY) and degassed prior to use. Isolated polysugar fractions were lyophilized, resuspended in a small volume of water, and incubated at 55°C overnight in order to decompose and remove any remaining ammonium bicarbonate. These steps were repeated until all of the salt was removed and the isolated sugars appeared as an anamorphous cotton-like material after the final lyophilization. The molecular sizes of biofilm matrix neutral carbohydrates were estimated using size exclusion column calibration with a set of *Leuconostoc* species dextran standards, which included 100-kDa, 70-kDa, 40-kDa, 25-kDa, and 6-kDa polymers.

To elucidate the structure of the isolated carbohydrates, we utilized a combination of one-dimensional (1D) ^1^H nuclear magnetic resonance (NMR) and 2D heteronuclear single quantum coherence (HSQC) NMR experiments as well as known chemical shift assignments characteristic of individual mannosyl and glucosyl motifs in mannan and glucan structures based on previously published studies (79–83). All data were collected at 70°C on a Bruker Biospin Avance III 500-MHz NMR spectrometer (Bruker BioSpin GmbH, Rheinstetten, Germany) equipped with a 5-mm triple resonance, cryogenic probe, CPTXI 500 H-C/N-D. One-dimensional spectra were collected with 32 acquisitions using a standard one-pulse experiment. The spectral width was 10 ppm centered at 4.7 ppm. The relaxation delay time was 2 s with an acquisition time of 3.3 s (32,768 data points). Thirty-two acquisitions were collected. Multiplicity-edited, phase-sensitive, echo-antiecho ^1^H HSQC spectra were obtained using four acquisitions per indirect time point with ^1^H decoupling during acquisition (84). Matched swept adiabatic ^13^C inversion pulses were used. The raw data matrix size was 2,048 × 128 blocks. Spectra were collected with a relaxation time delay of 2 s and an acquisition time of 0.2 s with sweep widths of 10 ppm (^1^H) and 65 ppm (^13^C), respectively. The centers of the spectra were 4.7 ppm for ^1^H and 82 ppm for ^13^C.

### Matrix carbohydrate fractionation and analysis for an MGCx.

Additional structural analysis was performed after a series of purification and fractionation steps to further ascertain the presence of a mannan-glucan complex. These steps include size exclusion chromatography followed by separation on an anion exchanger HiPrep 16/10 DEAE FF column (GE Healthcare Life Sciences). Neutral free carbohydrates were collected in flowthrough fractions, which were pooled and applied to gel filtration on a HighPrep 16/60 Sephacryl S-300 HR column (GE Healthcare Life Sciences), yielding 22 individual polysaccharide peaks, F1 to F22. The molecular weight of biofilm matrix neutral carbohydrates was determined using size exclusion column calibration with a set of *Leuconostoc* species dextran standards (polymers with molecular weights [in thousands] of 100, 70, 40, 25, and 6). Both high-molecular-weight (HMW) and low-molecular-weight (LMW) fractions were examined by GC for monosugar analysis. Matrix monosugar composition and quantification was performed on alditol acetate derivatives by GLC-FID (Shimadzu GC-2010 system; Shimadzu, Kyoto, Japan).

### *In vivo Candida* venous catheter biofilm model.

A jugular vein rat central venous catheter infection model was used for *in vivo* biofilm studies (53). *Candida* strains were grown to logarithmic phase in YPD at 30°C. After a 24-h conditioning period after catheter placement, infection was achieved by intraluminal instillation of 500 ml of C. albicans (10^6^ cells/ml). After an adherence period of 6 h, the 500-ml volume was withdrawn, and the catheter was flushed with heparinized saline. Following a 24-h incubation period, the catheters were removed, and the animals were prepared for SEM imaging as described above. The images were assembled using Adobe Photoshop 7.0.1.as described above. For drug treatment experiments, fluconazole (250 µg/ml) was instilled in the catheter after 24 h of biofilm growth. After a 24-h drug treatment period, the posttreatment viable burden of *Candida* biofilm on the catheter surface was measured by viable plate counts on Sabouraud’s dextrose agar (SDA) following removal of the biofilm by sonication and vortexing. We utilized three replicates for each condition.

### Sequestration of [^3^H]fluconazole in biofilms.

Radiolabeled fluconazole was used in an assay to assess drug retention in biofilms formed in six-well plates (85). Biofilms were grown for 48 h in six-well polystyrene plates as described above, washed, and then incubated with 8.48 × 10^5^ cpm of [^3^H]fluconazole (Moravek Biochemicals; 50 µM, 0.001 mCi/ml in ethanol) in RPMI-MOPS for 30 min at 37°C with orbital shaking at 50 rpm. Unlabeled fluconazole (20 µM) in RPMI-MOPS was added for an additional 15-min incubation period. After washing, biofilms and matrix were collected and isolated as described above. For a subset of biofilm cells, cells were disrupted by bead beating to yield cell wall and intracellular portions. Samples were added to a Tri-Carb 2100 TR liquid scintillation analyzer after adding ScintiSafe 30% liquid scintillation counting reagent (LSC) mixture to each sample fraction. The values for three technical replicates were averaged, the standard errors (SEs) were calculated, with values compared to the reference strain using pairwise comparisons with ANOVA by the Holm-Sidak method.

### Fluconazole-matrix interaction determination.

Interactions between the biofilm matrix and fluconazole were also probed using one-dimensional ^1^H NMR. Data were collected at 37°C on a Bruker Biospin Avance III 600-MHz NMR spectrometer (Bruker BioSpin GmbH) equipped with a 1.7-mm triple resonance, cryogenic probe, CPTXI 500 H-C/N-D. One-dimensional spectra were collected with 512 acquisitions using a one-pulse sequence experiment with water suppression and excitation sculpting with gradients (zgesgp). The spectral width was 16 ppm centered at 4.7 ppm. The relaxation delay time was 2 s. The approach was based on monitoring chemical shifts of fluconazole-specific protons in the presence and absence of the biofilm matrix under different pH conditions. In this study, all tested reactions were prepared in PBS (pH 7.2) and fluconazole was used at a constant concentration of 0.653 mM. In this drug-matrix system, interactions were represented by decreasing in signal intensities of the chemical shift peaks of protons present in fluconazole.
