# Supplementary material for: Conservation and Divergence in the Candida Species Biofilm Matrix Mannan-Glucan Complex Structure, Function, and Genetic Control
Source: mBio. 2018 Apr 3;9(2):e00451-18. doi: 10.1128/mBio.00451-18 (PMC5885036; doi:10.1128/mBio.00451-18)
Supplement: TABLE S1 [file mbo002183812st1.docx]

**Table S1. 2D HSQC NMR chemical shift assignment of the major spin systems found in *C. albicans*, *C. glabrata, C. tropicalis,* and *C. parapsilosis* (carbon chemical shifts in italics).** If a cell is left blank it means that the chemical shift was not found, either because the corresponding residue was absent or because of signal overlap.

***C. albicans***

| **No.** | **Residue** | **Chemical shift (ppm)** | | | | | | |
| --- | --- | --- | --- | --- | --- | --- | --- | --- |
|  |  | **1** | **2** | **3** | **4** | **5** | **6** | **6'** |
| **A** | α-1-2-Manα-1-3- | 5.36 | 4.08 | 3.98 | 3.76 | 3.76 | 3.86 | 3.73 |
|  |  | *103.2* | *81.2* | *72.9* | *69.2* | *76.1* | *63.8* | |
| **B** | α-1-2-Manα-1-2- | 5.26 | 4.09 | 3.94 | 3.74 | 3.69 | 3.86 | 3.73 |
|  |  | *103.3* | *81.2* | *72.9* | *69.2* | *76.0* | *63.8* | |
| **C** | α-1-2-Manα-1-2- | 5.25 | 4.10 | 3.95 | 3.73 | 3.75 | 3.87 | 3.74 |
|  |  | *103.3* | *81.2* | *72.9* | *69.2* | *76.0* | *64.0* | |
| **D** | α-1-2-Manα-1-2- | 5.24 | 4.08 | 3.89 | 3.70 | 3.72 | 3.86 | 3.73 |
|  |  | *103.3* | *81.2* | *73.1* | *69.2* | *76.1* | *63.8* | |
| **E** | β-1-2-Manα-1-2- | 5.13 | 4.24 | 3.85 | 3.61 | 3.72 | 3.86 | 3.73 |
|  |  | *102.8* | *80.5* | *73.1* | *69.7* | *76.1* | *63.8* | |
| **F** | 2,6-Manα-1-6- (l) |  |  |  |  |  |  |  |
|  |  |  |  |  |  |  |  | |
| **G** | 2,6-Manα-1-6- (l) |  |  |  |  |  |  |  |
|  |  |  |  |  |  |  |  | |
| **H** | 2,6-Manα-1-6- (b) | 5.07 | 4.00 | 3.93 | 3.78 | 3.80 | 3.95 | 3.74 |
|  |  | *101.0* | *73.0* | *73.0* | *69.3* | *73.6* | *68.4* | |
| **I** | Manα-1-2- | 5.03 | 4.05 | 3.83 | 3.64 | 3.74 | 3.86 | 3.73 |
|  |  | *104.9* | *72.9* | *73.2* | *69.7* | *76.1* | *63.8* | |
| **J** | 3-Manα-1-2- | 5.01 | 4.21 | 3.91 | 3.77 | 3.77 | 3.86 | 3.73 |
|  |  | *104.9* | *72.4* | *81.0* | *69.1* | *76.0* | *63.8* | |
| **K** | Manα-1-6- | 4.90 | 3.98 | 3.80 | 3.76 | 3.67 | 3.86 | 3.73 |
|  |  | *102.3* | *73.0* | *73.4* | *69.4* | *75.7* | *63.8* | |
| **L** | 6-Manα-1-6- | 4.89 | 3.99 | 3.83 | 3.76 | 3.78 | 3.95 | 3.74 |
|  |  | *102.3* | *73.0* | *73.3* | *69.4* | *73.9* | *68.4* | |
| **M** | β-1-2-Manβ-1-2- | 4.84 | 4.25 | 3.66 | 3.60 | 3.39 | 3.74 |  |
|  |  | *101.8* | *81.3* | *74.9* | *69.6* | *79.1* | *63.8* | |
| **N** | Manβ-1-2- | 4.83 | 4.13 | 3.60 | 3.58 | 3.35 | 3.89 | 3.74 |
|  |  | *103.7* | *73.0* | *75.6* | *69.6* | *79.1* | *63.8* | |
| **O** | Glcβ-1-6 | 4.72 | 3.35 | 3.50 | 3.44 | 3.47 | 3.84 | 3.75 |
|  |  | *105.6* | *76.1* | *78.5* | *72.5* | *78.5* | *63.6* | |
| **P** | β-1-6-Glcβ-1-6 | 4.51 | 3.32 | 3.47 | 3.44 | 3.60 | 4.20 | 3.85 |
|  |  | *105.6* | *75.8* | *78.5* | *72.4* | *77.6* | *71.4* | |

***C. glabrata***

| **No.** | **Residue** | **Chemical shift (ppm)** | | | | | | |
| --- | --- | --- | --- | --- | --- | --- | --- | --- |
|  |  | **1** | **2** | **3** | **4** | **5** | **6** | **6'** |
| **A** | α-1-2-Manα-1-3- |  |  |  |  |  |  |  |
|  |  |  |  |  |  |  |  | |
| **B** | α-1-2-Manα-1-2- |  |  |  |  |  |  |  |
|  |  |  |  |  |  |  |  | |
| **C** | α-1-2-Manα-1-2- | 5.25 | 4.09 | 3.89 | 3.71 | 3.75 | 3.87 | 3.74 |
|  |  | *103.2* | *81.2* | *73.1* | *69.8* | *76.0* | *64.0* | |
| **D** | α-1-2-Manα-1-2- |  |  |  |  |  |  |  |
|  |  |  |  |  |  |  |  | |
| **E** | β-1-2-Manα-1-2- | 5.16 | 4.26 | 3.85 | 3.76 |  |  |  |
|  |  | *102.6* | *80.6* | *72.2* | *69.4* |  |  | |
| **F** | 2,6-Manα-1-6- (l) | 5.10 | 4.00 |  |  |  |  |  |
|  |  | *100.9* | *81.4* |  |  |  |  | |
| **G** | 2,6-Manα-1-6- (l) | 5.06 | 4.02 |  |  |  |  |  |
|  |  | *101.1* | *81.4* |  |  |  |  | |
| **H** | 2,6-Manα-1-6- (b) | 5.05 | 4.00 | 3.90 |  | 3.76 | 3.95 | 3.74 |
|  |  | *101.0* | *81.4* | *73.2* |  | *73.9* | *68.3* | |
| **I** | Manα-1-2- | 5.04 | 4.05 | 3.83 | 3.64 | 3.74 | 3.87 | 3.74 |
|  |  | *104.9* | *73.0* | *73.2* | *69.7* | *76.0* | *64.0* | |
| **J** | 3-Manα-1-2- |  |  |  |  |  |  |  |
|  |  |  |  |  |  |  |  | |
| **K** | Manα-1-6- | 4.90 | 3.98 | 3.82 |  |  |  |  |
|  |  | *102.3* | *72.9* | *73.2* |  |  |  | |
| **L** | 6-Manα-1-6- | 4.88 | 3.97 | 3.82 |  |  | 4.00 | 3.68 |
|  |  | *102.2* | *72.9* | *73.2* |  |  | *68.5* | |
| **M** | β-1-2-Manβ-1-2- |  |  |  |  |  |  |  |
|  |  |  |  |  |  |  |  | |
| **N** | Manβ-1-2- |  |  |  |  |  |  |  |
|  |  |  |  |  |  |  |  | |
| **O** | Glcβ-1-6 |  |  |  |  |  |  |  |
|  |  |  |  |  |  |  |  | |
| **P** | β-1-6-Glcβ-1-6 |  |  |  |  |  |  |  |
|  |  |  |  |  |  |  |  | |

***C. tropicalis***

| **No.** | **Residue** | **Chemical shift (ppm)** | | | | | | |
| --- | --- | --- | --- | --- | --- | --- | --- | --- |
|  |  | **1** | **2** | **3** | **4** | **5** | **6** | **6'** |
| **A** | α-1-2-Manα-1-3- |  |  |  |  |  |  |  |
|  |  |  |  |  |  |  |  | |
| **B** | α-1-2-Manα-1-2- | 5.27 | 4.09 | 3.94 | 3.72 | 3.72 | 3.86 | 3.73 |
|  |  | *103.3* | *81.2* | *73.0* | *69.2* | *76.1* | *63.8* | |
| **C** | α-1-2-Manα-1-2- |  |  |  |  |  |  |  |
|  |  |  |  |  |  |  |  | |
| **D** | α-1-2-Manα-1-2- | 5.25 | 4.08 | 3.89 | 3.70 | 3.72 | 3.86 | 3.73 |
|  |  | *103.3* | *81.2* | *73.1* | *69.2* | *76.1* | *63.8* | |
| **E** | β-1-2-Manα-1-2- | 5.13 | 4.24 | 3.85 | 3.61 | 3.72 | 3.86 | 3.73 |
|  |  | *102.8* | *80.5* | *73.1* | *69.7* | *76.1* | *63.8* | |
| **F** | 2,6-Manα-1-6- (l) |  |  |  |  |  |  |  |
|  |  |  |  |  |  |  |  | |
| **G** | 2,6-Manα-1-6- (l) |  |  |  |  |  |  |  |
|  |  |  |  |  |  |  |  | |
| **H** | 2,6-Manα-1-6- (b) | 5.07 | 4.00 | 3.93 | 3.78 | 3.80 | 3.95 | 3.74 |
|  |  | *101.0* | *73.0* | *73.0* | *69.3* | *73.6* | *68.4* | |
| **I** | Manα-1-2- | 5.03 | 4.05 | 3.83 | 3.64 | 3.74 | 3.86 | 3.73 |
|  |  | *104.9* | *72.9* | *73.2* | *69.7* | *76.1* | *63.8* | |
| **J** | 3-Manα-1-2- |  |  |  |  |  |  |  |
|  |  |  |  |  |  |  |  | |
| **K** | Manα-1-6- | 4.90 | 3.98 | 3.80 |  |  |  |  |
|  |  | *102.3* | *73.0* | *73.4* |  |  |  | |
| **L** | 6-Manα-1-6- | 4.89 | 3.99 |  |  |  |  |  |
|  |  | *102.3* | *73.0* |  |  |  |  | |
| **M** | β-1-2-Manβ-1-2- | 4.83 | 4.25 |  |  |  |  |  |
|  |  | *101.7* | *81.2* |  |  |  |  | |
| **N** | Manβ-1-2- | 4.82 | 4.14 |  |  |  |  |  |
|  |  | *103.7* | *73.1* |  |  |  |  | |
| **O** | Glcβ-1-6 | 4.72 | 3.35 | 3.50 | 3.44 | 3.47 | 3.84 | 3.75 |
|  |  |  | *76.1* | *78.5* | *72.5* | *78.5* | *63.6* | |
| **P** | β-1-6-Glcβ-1-6 | 4.51 | 3.32 | 3.48 | 3.44 | 3.61 | 4.21 | 3.85 |
|  |  | *105.6* | *76.0* | *78.5* | *72.5* | *77.7* | *71.6* | |

***C. parapsilosis***

| **No.** | **Residue** | **Chemical shift (ppm)** | | | | | | |
| --- | --- | --- | --- | --- | --- | --- | --- | --- |
|  |  | **1** | **2** | **3** | **4** | **5** | **6** | **6'** |
| **A** | α-1-2-Manα-1-3- | 5.36 | 4.08 |  |  |  |  |  |
|  |  | *103.3* | *81.3* |  |  |  |  | |
| **B** | α-1-2-Manα-1-2- | 5.27 | 4.09 | 3.92 | 3.72 | 3.72 | 3.86 | 3.73 |
|  |  | *103.4* | *81.2* | *73.0* | *69.5* | *76.1* | *63.8* | |
| **C** | α-1-2-Manα-1-2- |  |  |  |  |  |  |  |
|  |  |  |  |  |  |  |  | |
| **D** | α-1-2-Manα-1-2- | 5.25 | 4.08 | 3.89 | 3.69 | 3.72 | 3.86 | 3.73 |
|  |  | *103.4* | *81.2* | *72.9* | *69.9* | *76.1* | *63.8* | |
| **E** | β-1-2-Manα-1-2- |  |  |  |  |  |  |  |
|  |  |  |  |  |  |  |  | |
| **F** | 2,6-Manα-1-6- (l) |  |  |  |  |  |  |  |
|  |  |  |  |  |  |  |  | |
| **G** | 2,6-Manα-1-6- (l) |  |  |  |  |  |  |  |
|  |  |  |  |  |  |  |  | |
| **H** | 2,6-Manα-1-6- (b) | 5.08 | 3.98 | 3.92 | 3.78 | 3.78 | 3.95 | 3.74 |
|  |  | *101.1* | *81.6* | *73.0* | *69.4* | *73.8* | *68.4* | |
| **I** | Manα-1-2- | 5.04 | 4.05 | 3.83 | 3.64 | 3.74 | 3.86 | 3.73 |
|  |  | *104.9* | *72.8* | *73.4* | *69.8* | *76.1* | *63.8* | |
| **J** | 3-Manα-1-2- | 5.02 | 4.19 | 3.92 | 3.78 | 3.77 | 3.85 | 3.74 |
|  |  | *104.9* | *72.7* | *81.2* | *69.5* | *76.0* | *63.8* | |
| **K** | Manα-1-6- | 4.90 | 3.98 | 3.80 | 3.67 | 3.75 | 3.86 | 3.73 |
|  |  | *102.4* | *72.9* | *73.9* | *69.7* | *76.0* | *63.8* | |
| **L** | 6-Manα-1-6- | 4.88 | 3.97 | 3.82 | 3.78 | 3.78 | 3.95 | 3.74 |
|  |  | *102.2* | *72.9* | *73.6* | *69.4* | *73.8* | *68.4* | |
| **M** | β-1-2-Manβ-1-2- |  |  |  |  |  |  |  |
|  |  |  |  |  |  |  |  | |
| **N** | Manβ-1-2- |  |  |  |  |  |  |  |
|  |  |  |  |  |  |  |  | |
| **O** | Glcβ-1-6 | 4.70 | 3.35 | 3.50 | 3.41 | 3.47 | 3.83 | 3.75 |
|  |  | *105.7* | *75.9* | *78.5* | *72.4* | *78.5* | *63.4* | |
| **P** | β-1-6-Glcβ-1-6 | 4.50 | 3.31 | 3.49 | 3.41 | 3.61 | 4.21 | 3.85 |
|  |  | *105.7* | *75.8* | *78.5* | *72.4* | *77.6* | *71.6* | |
