# Supplementary figures and images for: Conservation and Divergence in the Candida Species Biofilm Matrix Mannan-Glucan Complex Structure, Function, and Genetic Control
Source: mBio. 2018 Apr 3;9(2):e00451-18. doi: 10.1128/mBio.00451-18 (PMC5885036; doi:10.1128/mBio.00451-18)

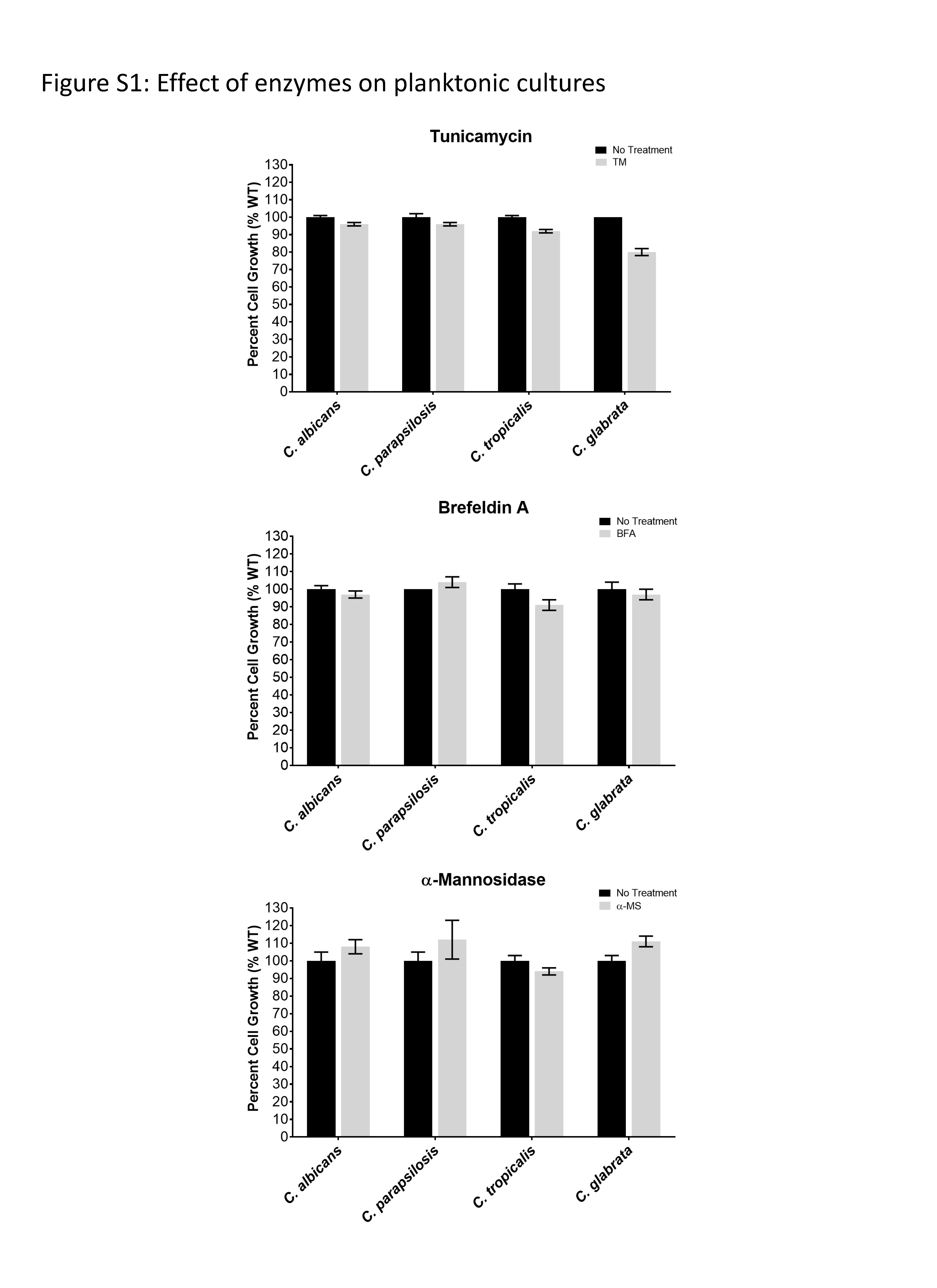

Supplement: FIG S1 [file mbo002183812sf1.tif]

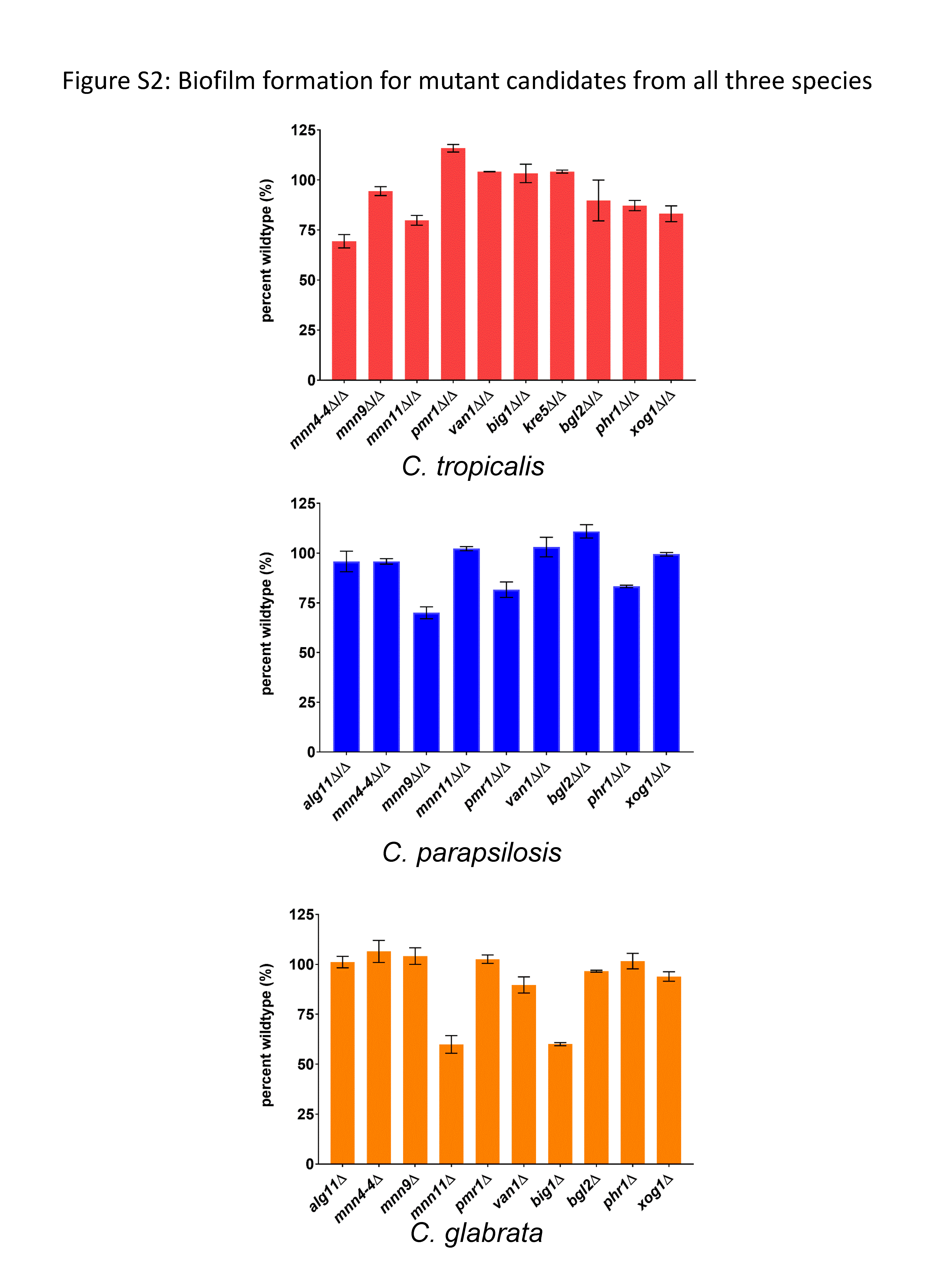

Supplement: FIG S2 [file mbo002183812sf2.tif]

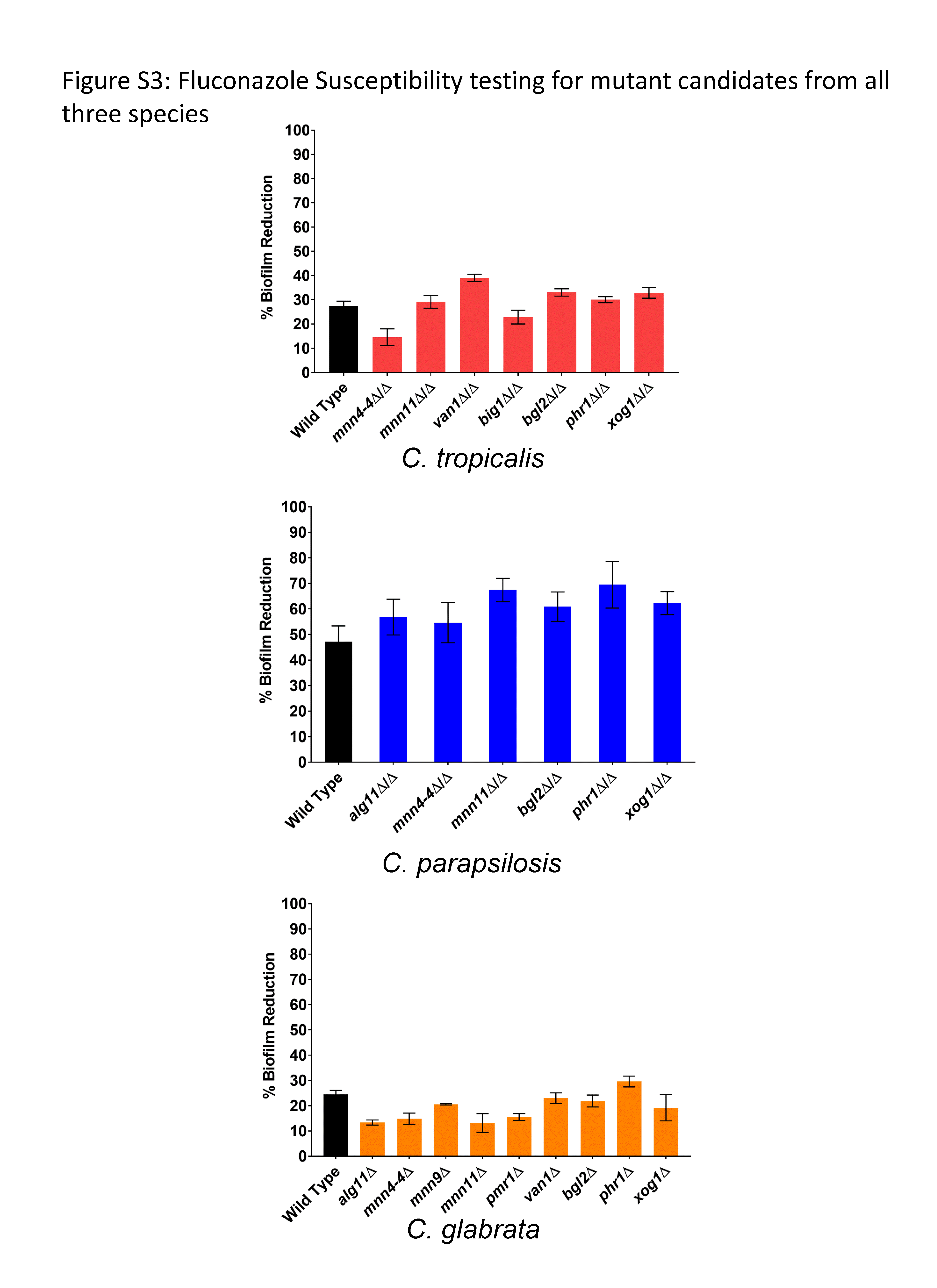

Supplement: FIG S3 [file mbo002183812sf3.tif]

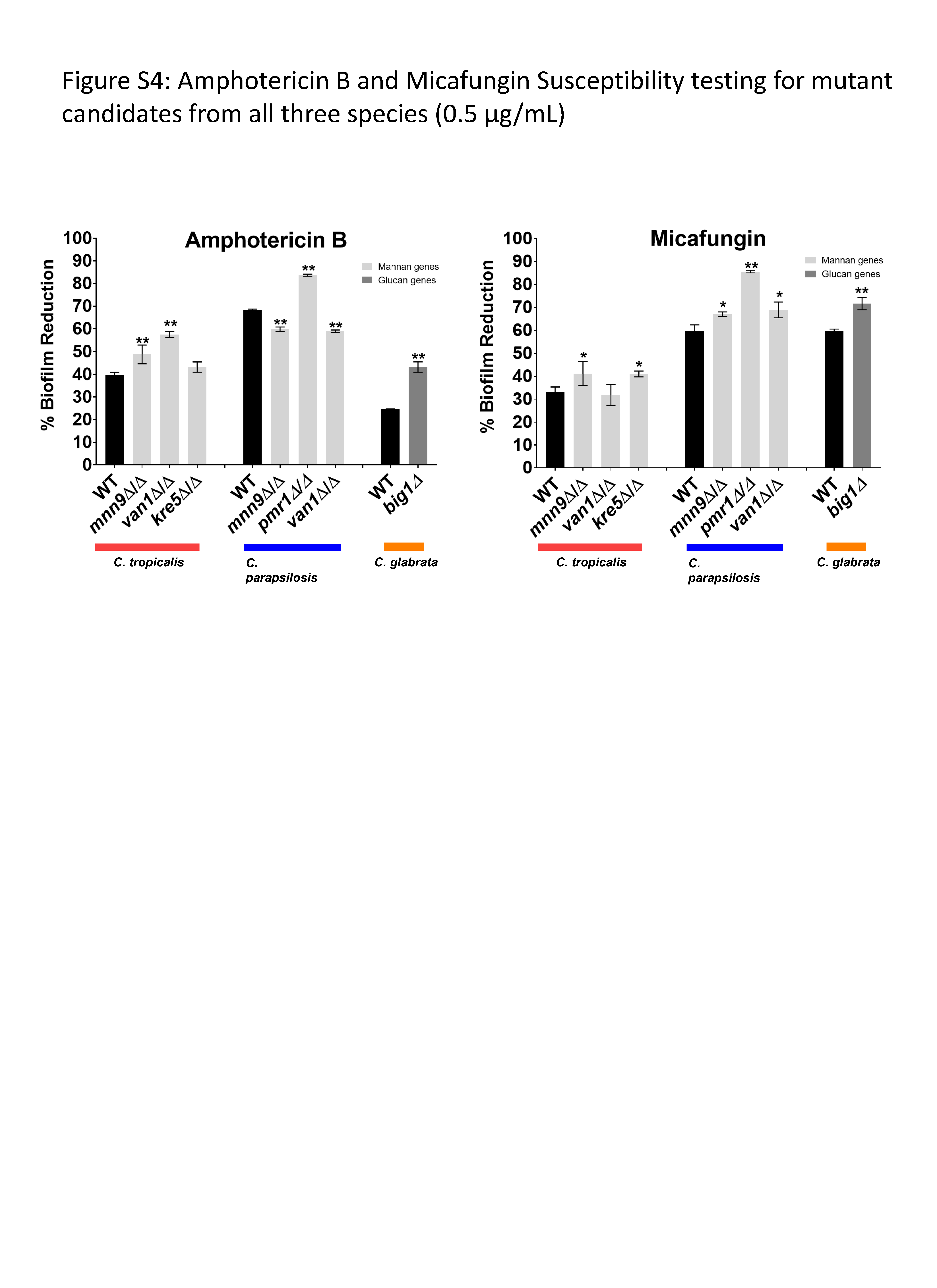

Supplement: FIG S4 [file mbo002183812sf4.tif]

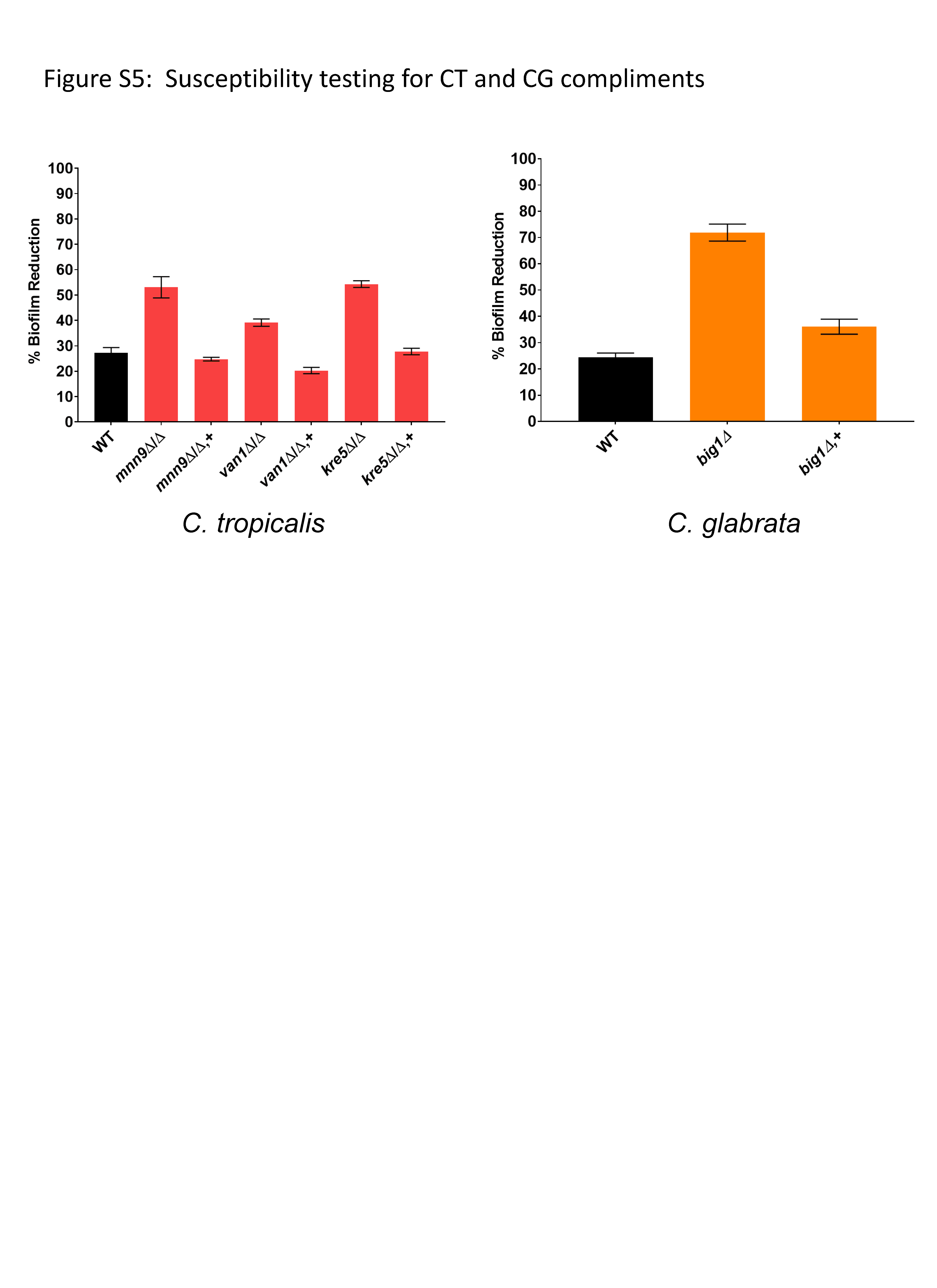

Supplement: FIG S5 [file mbo002183812sf5.tif]

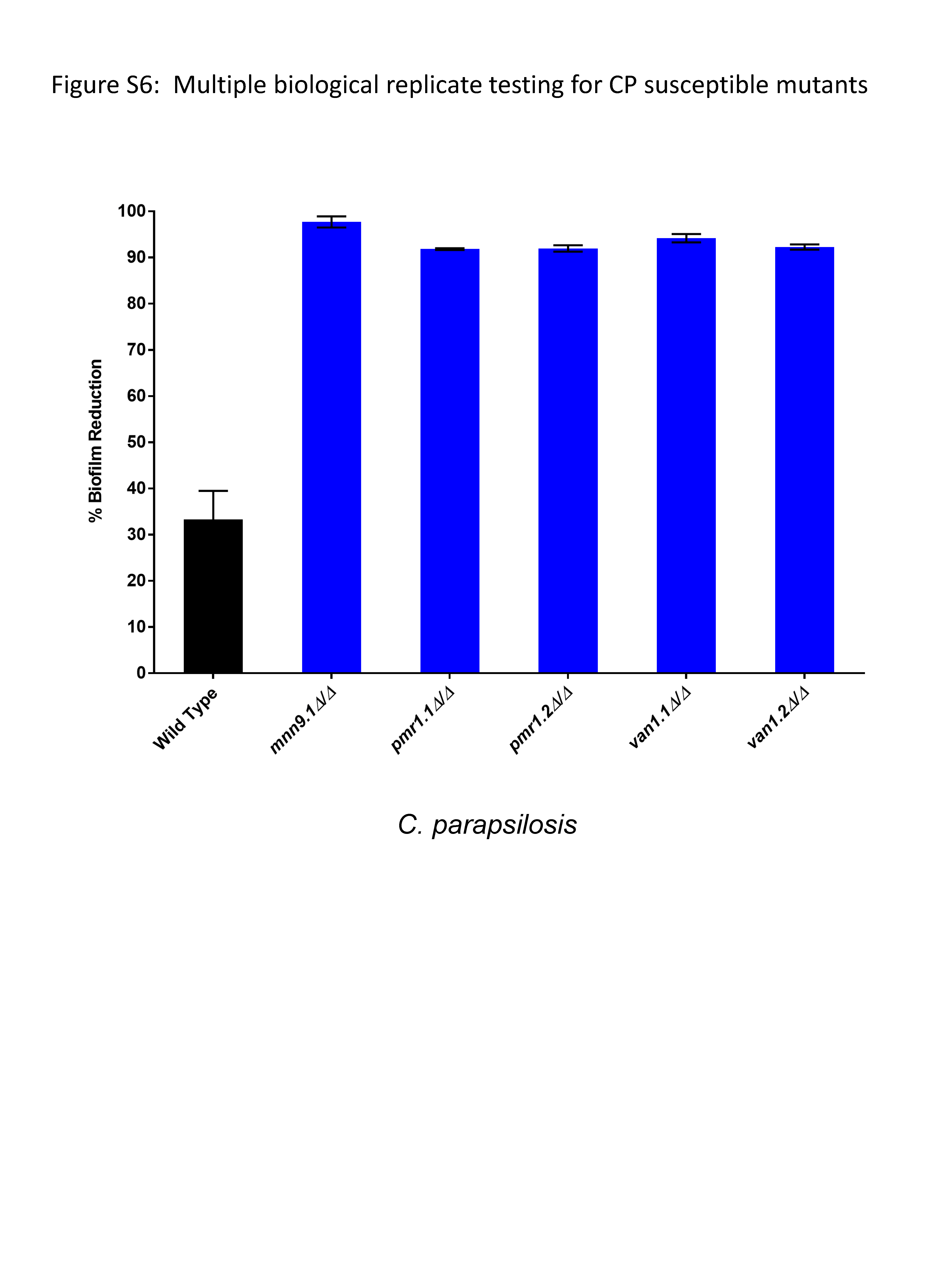

Supplement: FIG S6 [file mbo002183812sf6.tif]
