# Supplementary material for: Conservation and Divergence in the Candida Species Biofilm Matrix Mannan-Glucan Complex Structure, Function, and Genetic Control
Source: mBio. 2018 Apr 3;9(2):e00451-18. doi: 10.1128/mBio.00451-18 (PMC5885036; doi:10.1128/mBio.00451-18)
Supplement: TABLE S2 [file mbo002183812st2.docx]

**Table S2. *C. tropicalis, C. parapsilosis,* and *C. glabrata* mutant strains developed in this study.**

| **Gene** | **Strain** | **Genotype** | **Source** |
| --- | --- | --- | --- |
| Reference | CAY2597 | *C. tropicalis* wild type strain | (Ref 5) |
| Reference | CAY3764 | *his1Δ::FRT/his1Δ::FRT, leu2Δ::FRT/leu2Δ::FRT* | (Ref 6) |
| *big1* ^-/-^ | EGD192 | *his1Δ::FRT/his1Δ::FRT, leu2Δ::FRT/leu2Δ::FRT, big1::C.m LEU2/big1::C.d HIS1* | This study |
| *bgl2* ^-/-^ | EGD151 | *his1Δ::FRT/his1Δ::FRT, leu2Δ::FRT/leu2Δ::FRT, bgl2::C.m LEU2/bgl2::C.d HIS1* | This study |
| *bgl2* ^-/-^ | EGD152 | *his1Δ::FRT/his1Δ::FRT, leu2Δ::FRT/leu2Δ::FRT, bgl2::C.m LEU2/bgl2::C.d HIS1* | This study |
| *kre5* ^-/-^ | EGD186 | *his1Δ::FRT/his1Δ::FRT, leu2Δ::FRT/leu2Δ::FRT, kre5::C.m LEU2/kre5::C.d HIS1* | This study |
| *kre5* ^-/-^ | EGD187 | *his1Δ::FRT/his1Δ::FRT, leu2Δ::FRT/leu2Δ::FRT, kre5::C.m LEU2/kre5::C.d HIS1* | This study |
| *kre5* ^-/-^,+ | URZ492 | *his1Δ::FRT/his1Δ::FRT, leu2Δ::FRT/leu2Δ::FRT, kre5::C.m LEU2/kre5::C.d HIS1,C.m leu2::KRE5-NAT1* | This study |
| *mnn4-4* ^-/-^ | EGD155 | *his1Δ::FRT/his1Δ::FRT, leu2Δ::FRT/leu2Δ::FRT, mnn4-4::C.m LEU2/mnn4-4::C.d HIS1* | This study |
| *mnn4-4* ^-/-^ | EGD157 | *his1Δ::FRT/his1Δ::FRT, leu2Δ::FRT/leu2Δ::FRT, mnn4-4::C.m LEU2/mnn4-4::C.d HIS1* | This study |
| *mnn9* ^-/-^ | EGD179 | *his1Δ::FRT/his1Δ::FRT, leu2Δ::FRT/leu2Δ::FRT, mnn9::C.m LEU2/mnn9::C.d HIS1* | This study |
| *mnn9* ^-/-^ | EGD182 | *his1Δ::FRT/his1Δ::FRT, leu2Δ::FRT/leu2Δ::FRT, mnn9::C.m LEU2/mnn9::C.d HIS1* | This study |
| *mnn9* ^-/-^,+ | URZ494 | *his1Δ::FRT/his1Δ::FRT, leu2Δ::FRT/leu2Δ::FRT, mnn9::C.m LEU2/mnn9::C.d HIS1,C.m leu2::MNN9-NAT1* | This study |
| *mnn11* ^-/-^ | EGD159 | *his1Δ::FRT/his1Δ::FRT, leu2Δ::FRT/leu2Δ::FRT, mnn11::C.m LEU2/mnn11::C.d HIS1* | This study |
| *mnn11* ^-/-^ | EGD160 | *his1Δ::FRT/his1Δ::FRT, leu2Δ::FRT/leu2Δ::FRT, mnn11::C.m LEU2/mnn11::C.d HIS1* | This study |
| *phr1* ^-/-^ | EGD171 | *his1Δ::FRT/his1Δ::FRT, leu2Δ::FRT/leu2Δ::FRT, phr1::C.m LEU2/phr1::C.d HIS1* | This study |
| *phr1* ^-/-^ | EGD173 | *his1Δ::FRT/his1Δ::FRT, leu2Δ::FRT/leu2Δ::FRT, phr1::C.m LEU2/phr1::C.d HIS1* | This study |
| *pmr1* ^-/-^ | EGD163 | *his1Δ::FRT/his1Δ::FRT, leu2Δ::FRT/leu2Δ::FRT, pmr1::C.m LEU2/pmr1::C.d HIS1* | This study |
| *pmr1* ^-/-^ | EGD165 | *his1Δ::FRT/his1Δ::FRT, leu2Δ::FRT/leu2Δ::FRT, pmr1::C.m LEU2/pmr1::C.d HIS1* | This study |
| *van1* ^-/-^ | EGD168 | *his1Δ::FRT/his1Δ::FRT, leu2Δ::FRT/leu2Δ::FRT, van1::C.m LEU2/van1::C.d HIS1* | This study |
| *van1* ^-/-^ | EGD169 | *his1Δ::FRT/his1Δ::FRT, leu2Δ::FRT/leu2Δ::FRT, van1::C.m LEU2/van1::C.d HIS1* | This study |
| *van1* ^-/-^,+ | URZ497 | *his1Δ::FRT/his1Δ::FRT, leu2Δ::FRT/leu2Δ::FRT, van1::C.m LEU2/van1::C.d HIS1, C.d his1::VAN1-NAT1* | This study |
| *xog1* ^-/-^ | EGD176 | *his1Δ::FRT/his1Δ::FRT, leu2Δ::FRT/leu2Δ::FRT, xog1::C.m LEU2/xog1::C.d HIS1* | This study |
| *xog1* ^-/-^ | EGD177 | *his1Δ::FRT/his1Δ::FRT, leu2Δ::FRT/leu2Δ::FRT, xog1::C.m LEU2/xog1::C.d HIS1* | This study |
| **Gene** | **Strain** | **Genotype** | **Source** |
| Reference | CLIB214 | *C. parapsilosis* wild type strain |  |
| Reference | CPL2H1 | *leu2Δ::FRT/leu2Δ::FRT, his1Δ::FRT/his1Δ::FRT* |  |
| *alg11* ^-/-^ | EGD136 | *leu2Δ::FRT/leu2Δ::FRT, his1Δ::FRT/his1Δ::FRT, alg11::C.m LEU2/alg11::C.d HIS1* | This study |
| *bgl2* ^-/-^ | EGD146 | *leu2Δ::FRT/leu2Δ::FRT, his1Δ::FRT/his1Δ::FRT, bgl2::C.m LEU2/bgl2::C.d HIS1* | This study |
| *bgl2* ^-/-^ | EGD147 | *leu2Δ::FRT/leu2Δ::FRT, his1Δ::FRT/his1Δ::FRT, bgl2::C.m LEU2/bgl2::C.d HIS1* | This study |
| *mnn4-4* ^-/-^ | EGD148 | *leu2Δ::FRT/leu2Δ::FRT, his1Δ::FRT/his1Δ::FRT, mnn4-4::C.m LEU2/mnn4-4::C.d HIS1* | This study |
| *mnn4-4* ^-/-^ | EGD149 | *leu2Δ::FRT/leu2Δ::FRT, his1Δ::FRT/his1Δ::FRT, mnn4-4::C.m LEU2/mnn4-4::C.d HIS1* | This study |
| *mnn4-4* ^-/-^,+ | URZ488 | *leu2Δ::FRT/leu2Δ::FRT, his1Δ::FRT/his1Δ::FRT, mnn4-4::C.m LEU2/mnn4-4::C.d HIS1, C.m leu2::MNN4-4-NAT1* | This study |
| *mnn9* ^-/-^ | EGD193 | *leu2Δ::FRT/leu2Δ::FRT, his1Δ::FRT/his1Δ::FRT, mnn9::C.m LEU2/mnn9::C.d HIS1* | This study |
| *mnn9* ^-/-^ | EGD194 | *leu2Δ::FRT/leu2Δ::FRT, his1Δ::FRT/his1Δ::FRT, mnn9::C.m LEU2/mnn9::C.d HIS1* | This study |
| *mnn9* ^-/-^,+ | URZ485 | *leu2Δ::FRT/leu2Δ::FRT, his1Δ::FRT/his1Δ::FRT, mnn9::C.m LEU2/mnn9::C.d HIS1, C.d his1::MNN9-NAT1* | This study |
| *mnn11* ^-/-^ | EGD144 | *leu2Δ::FRT/leu2Δ::FRT, his1Δ::FRT/his1Δ::FRT, mnn11::C.m LEU2/mnn11::C.d HIS1* | This study |
| *mnn11* ^-/-^ | EGD145 | *leu2Δ::FRT/leu2Δ::FRT, his1Δ::FRT/his1Δ::FRT, mnn11::C.m LEU2/mnn11::C.d HIS1* | This study |
| *mnn11* ^-/-^,+ | URZ486 | *leu2Δ::FRT/leu2Δ::FRT, his1Δ::FRT/his1Δ::FRT, mnn11::C.m LEU2/mnn11::C.d HIS1, C.m leu2::MNN11-NAT1* | This study |
| *phr1* ^-/-^ | EGD188 | *leu2Δ::FRT/leu2Δ::FRT, his1Δ::FRT/his1Δ::FRT, phr1::C.m LEU2/phr1::C.d HIS1* | This study |
| *phr1* ^-/-^ | EGD189 | *leu2Δ::FRT/leu2Δ::FRT, his1Δ::FRT/his1Δ::FRT, phr1::C.m LEU2/phr1::C.d HIS1* | This study |
| *phr1* ^-/-^,+ | URZ491 | *leu2Δ::FRT/leu2Δ::FRT, his1Δ::FRT/his1Δ::FRT, phr1::C.m LEU2/phr1::C.d HIS1, C.m leu2::PHR1-NAT1* | This study |
| *pmr1.1* ^-/-^ | EGD141 | *leu2Δ::FRT/leu2Δ::FRT, his1Δ::FRT/his1Δ::FRT, pmr1::C.m LEU2/pmr1::C.d HIS1* | This study |
| *pmr1.2* ^-/-^ | EGD142 | *leu2Δ::FRT/leu2Δ::FRT, his1Δ::FRT/his1Δ::FRT, pmr1::C.m LEU2/pmr1::C.d HIS1* | This study |
| *pmr1* ^-/-^,+ | URZ520 | *leu2Δ::FRT/leu2Δ::FRT, his1Δ::FRT/his1Δ::FRT, pmr1::C.m LEU2/pmr1::C.d HIS1, C.m leu2::PMR1-NAT1* | This study |
| *van1* ^-/-^ | EGD184 | *leu2Δ::FRT/leu2Δ::FRT, his1Δ::FRT/his1Δ::FRT, van1::C.m LEU2/van1::C.d HIS1* | This study |
| *van1* ^-/-^ | EGD185 | *leu2Δ::FRT/leu2Δ::FRT, his1Δ::FRT/his1Δ::FRT, van1::C.m LEU2/van1::C.d HIS1* | This study |
| *van1* ^-/-^,+ | URZ510 | *leu2Δ::FRT/leu2Δ::FRT, his1Δ::FRT/his1Δ::FRT, van1::C.m LEU2/van1::C.d HIS1, C.m leu2::VAN1-NAT1* | This study |
| *xog1* ^-/-^ | EGD150 | *leu2Δ::FRT/leu2Δ::FRT, his1Δ::FRT/his1Δ::FRT, xog1::C.m LEU2/xog1::C.d HIS1* | This study |
| *xog1* ^-/-^,+ | URZ499 | *leu2Δ::FRT/leu2Δ::FRT, his1Δ::FRT/his1Δ::FRT, xog1::C.m LEU2/xog1::C.d HIS1, C.m leu2::XOG1-NAT1* | This study |

| **Gene** | **Strain** | **Genotype** | **Source** |
| --- | --- | --- | --- |
| Reference | ATCC2001 | *C. glabrata* wild type strain |  |
| Reference | HTL | *his3*∆::FRT, *leu2*∆::FRT, *trp1*∆::FRT |  |
| *alg11* Δ | EGD125 | *his3*∆::FRT, *leu2*∆::FRT, *trp1*∆::FRT, *alg11*∆::NAT1 | This study |
| *alg11* Δ | EGD126 | *his3*∆::FRT, *leu2*∆::FRT, *trp1*∆::FRT, *alg11*∆::NAT1 | This study |
| *bgl2* Δ | EGD127 | *his3*∆::FRT, *leu2*∆::FRT, *trp1*∆::FRT, *bgl2*∆::NAT1 | This study |
| *big1* Δ | EGD129 | *his3*∆::FRT, *leu2*∆::FRT, *trp1*∆::FRT, *big1*∆::NAT1 | This study |
| *big1* Δ | EGD130 | *his3*∆::FRT, *leu2*∆::FRT, *trp1*∆::FRT, *big1*∆::NAT1 | This study |
| *big1* Δ,+ | URZ508 | *his3*∆::FRT, *leu2*∆::FRT, *trp1*∆::FRT, *big1*∆::NAT1 / NAT1::BIG1-HygB | This study |
| *mnn4-4* Δ | EGD121 | *his3*∆::FRT, *leu2*∆::FRT, *trp1*∆::FRT,*mnn4-4*∆::NAT1 | This study |
| *mnn4-4* Δ | EGD122 | *his3*∆::FRT, *leu2*∆::FRT, *trp1*∆::FRT,*mnn4-4*∆::NAT1 | This study |
| *mnn9* Δ | EGD128 | *his3*∆::FRT, *leu2*∆::FRT, *trp1*∆::FRT, *mnn9*∆::NAT1 | This study |
| *mnn11* Δ | EGD124 | *his3*∆::FRT, *leu2*∆::FRT, *trp1*∆::FRT, *mnn11*∆::NAT1 | This study |
| *phr1* Δ | EGD131 | *his3*∆::FRT, *leu2*∆::FRT, *trp1*∆::FRT, *phr1*∆::NAT1 | This study |
| *phr1* Δ | EGD132 | *his3*∆::FRT, *leu2*∆::FRT, *trp1*∆::FRT, *phr1*∆::NAT1 | This study |
| *pmr1* Δ | EGD143 | *his3*∆::FRT, *leu2*∆::FRT, *trp1*∆::FRT, *pmr1*∆::NAT1 | This study |
| *van1* Δ | EGD137 | *his3*∆::FRT, *leu2*∆::FRT, *trp1*∆::FRT, *van1*∆::NAT1 | This study |
| *van1* Δ | EGD138 | *his3*∆::FRT, *leu2*∆::FRT, *trp1*∆::FRT, *van1*∆::NAT1 | This study |
| *xog1* Δ | EGD134 | *his3*∆::FRT, *leu2*∆::FRT, *trp1*∆::FRT, *xog1*∆::NAT1 | This study |
| *xog1* Δ | EGD135 | *his3*∆::FRT, *leu2*∆::FRT, *trp1*∆::FRT, *xog1*∆::NAT1 | This study |
