# Supplementary material for: Conservation and Divergence in the Candida Species Biofilm Matrix Mannan-Glucan Complex Structure, Function, and Genetic Control
Source: mBio. 2018 Apr 3;9(2):e00451-18. doi: 10.1128/mBio.00451-18 (PMC5885036; doi:10.1128/mBio.00451-18)
Supplement: TABLE S3 [file mbo002183812st3.docx]

**Table S3. Primers for *C. tropicalis, C. parapsilosis* and *C. glabrata* Mutant Strain Creation**

***C. tropicalis***

| **Gene** | **Function** | **Primers** |
| --- | --- | --- |
| *BGL2* | Knockout | Upstream F: 5’ – GCCTTACGTTTTTGATCTTATT  Upstream R: 5’ – CACGGCGCGCCTAGCAGCGGGACCAAAAATGATAACGAGAGT  Auxotrophic marker F: 5’ – CCGCTGCTAGGCGCGCCGTGACCAGTGTGATGGATATCTGC  Auxotrophic marker R: 5’ – GCAGGGATGCGGCCGCTGACAGCTCGGATCCACTAGTAACG  Downstream F: 5’ – GTCAGCGGCCGCATCCCTGCTCGTTTTAGAATATCCTATTTGC  Downstream R: 5’ – GTATTTTCCATGTTGCCATCTA  Nested Fusion F: 5’ – GTCATGGTGAAGAGAAGTTTTT  Nested Fusion R: 5’ – TAACTAATCCCTGGTAATTTGG  Internal Check F: 5’ – GCAATTCAAATACTTAGCAGGT  Internal Check R: 5’ – TTTCTAGAAGAATCCCAAACAC  Upstream Check F: 5’ – TAGTATGGCAATGGTGTCAG  Downstream Check R: 5’ – ATTGAGCCAGTTCAAAATGT  His Upstream Check F: 5` – AAAATCAATGGGCATTCTCG  His Downstream Check R: 5` – TGGGAAGCAGACATTCAACA  Leu Upstream Check F: 5` – GAAGTTGGTGACGCGATTGT  Leu Downstream Check F: 5` – TTCCCCTTCAATGTATGCAA |
| *BIG1* | Knockout | Upstream F: 5’ – CTAGTGTTTGTCGGTGTGTG  Upstream R: 5’ – CACGGCGCGCCTAGCAGCGGTTTTCTTCCTCGTTTTTCAA  Auxotrophic marker F: 5’ – CCGCTGCTAGGCGCGCCGTGACCAGTGTGATGGATATCTGC  Auxotrophic marker R: 5’ – GCAGGGATGCGGCCGCTGACAGCTCGGATCCACTAGTAACG  Downstream F: 5’ – GTCAGCGGCCGCATCCCTGCTTTATCTTATTTGTCACCACCA  Downstream R: 5’ – GGTGTTGAATATCAACCAACTT  Nested Fusion F: 5’ – TTTCTCTTCAACTCCTGTTTCT  Nested Fusion R: 5’ – GAACAAGTGGTGTTGACAGTT  Internal Check F: 5’ – TAGTCACTTGTCGAGTTACACC  Internal Check R: 5’ – AAAGTCTTCAAAAAGGATACGA  Upstream Check F: 5’ – GGTATCATCACCAGCTTTGT  Downstream Check R: 5’ – TTCAGAAGAATCAGGGTCTG  His Upstream Check F: 5` – AAAATCAATGGGCATTCTCG  His Downstream Check R: 5` – TGGGAAGCAGACATTCAACA  Leu Upstream Check F: 5` – GAAGTTGGTGACGCGATTGT  Leu Downstream Check F: 5` – TTCCCCTTCAATGTATGCAA |
| *KRE5* | Knockout | Upstream F: 5’ – ATGAAATGTTTCCTAAAAATGC  Upstream R: 5’ – CACGGCGCGCCTAGCAGCGGATCGTTATATGTTTCGGTTTTC  Auxotrophic marker F: 5’ – CCGCTGCTAGGCGCGCCGTGACCAGTGTGATGGATATCTGC  Auxotrophic marker R: 5’ – GCAGGGATGCGGCCGCTGACAGCTCGGATCCACTAGTAACG  Downstream F: 5’ – GTCAGCGGCCGCATCCCTGCCCTGAATCGGAGTTTTTATTAG  Downstream R: 5’ – TACGTAATTTTGAACACCAAGA  Nested Fusion F: 5’ – GAATGAAATTGTGGGTACTTTT  Nested Fusion R: 5’ – TTTCAATTCATCTGTTTCTTCA  Internal Check F: 5’ – TACTAATCGTGAGGAGGCTTAT  Internal Check R: 5’ – AGTTTGTCAAAGTGGTTAGCTT  Upstream Check F: 5’ – CGACCACATCACAATGATAG  Downstream Check R: 5’ – TCAGCATCGTAAAACATTTG  His Upstream Check F: 5` – AAAATCAATGGGCATTCTCG  His Downstream Check R: 5` – TGGGAAGCAGACATTCAACA  Leu Upstream Check F: 5` – GAAGTTGGTGACGCGATTGT  Leu Downstream Check F: 5` – TTCCCCTTCAATGTATGCAA |
| *KRE5* | Compliment | Upstream/ORF F: 5’ – ATGCGAAATCATTACTATACGA  Upstream/ORF R: 5’ – CACGGCGCGCCTAGCAGCGGATTCAGGAATAGCACGTGAA  Antibiotic marker F: 5’ – CCGCTGCTAGGCGCGCCGTGGATATCAAGCTTGCCTCGTCC  Antibiotic marker R: 5’ – GCAGGGATGCGGCCGCTGACTTACTTTCTGCGCACTTAACTTC  Downstream F: 5’ – GTCAGCGGCCGCATCCCTGCGCACGTGCACTTGTTAGATA  Downstream R: 5’ – TTTTACGTAATTTTGAACACCA  Nested Fusion F: 5’ – ATGAAATGTTTCCTAAAAATGC  Nested Fusion R: 5’ – TTTCAATTCATCTGTTTCTTCA  Internal Check F: 5’ – TACTAATCGTGAGGAGGCTTAT  Internal Check R: 5’ – AGTTTGTCAAAGTGGTTAGCTT  Upstream Check F: 5’ – GGTGGCACCGATAATACAT  Downstream Check R: 5’ – ATCGTTATATGTTTCGGTTTTC  Nourseothricin Upstream Check F: 5` – TGGTTTCGTTGTTGTTTCTTAT  Nourseothricin Downstream Check R: 5` – TTATGAGCTTGATCCAACTTCT |
| *MNN4-4* | Knockout | Upstream F: 5’ – CACGACCTATTTCCTAATAACA  Upstream R: 5’ – CACGGCGCGCCTAGCAGCGGATGTGAAATGTGAAATGCTAAA  Auxotrophic marker F: 5’ – CCGCTGCTAGGCGCGCCGTGACCAGTGTGATGGATATCTGC  Auxotrophic marker R: 5’ – GCAGGGATGCGGCCGCTGACAGCTCGGATCCACTAGTAACG  Downstream F: 5’ – GTCAGCGGCCGCATCCCTGCACATAAATCTCTTATTAACAAACAAAC  Downstream R: 5’ – TGTTAGAAAAGAATTAACAGTTAGTGA  Nested Fusion F: 5’ – CCATGGTACATAAACAATTGAA  Nested Fusion R: 5’ – AAAGATCCAGAAAAGAGACAAA  Internal Check F: 5’ – AAATGGGAAGAATTGTTTAATG  Internal Check R: 5’ – TCACTTGGATTTTCAACAACTA  Upstream Check F: 5’ – TTATGAAATTAACACAAACATGG  Downstream Check R: 5’ – TTCAATGTGAAAGATCAGGA  His Upstream Check F: 5` – AAAATCAATGGGCATTCTCG  His Downstream Check R: 5` – TGGGAAGCAGACATTCAACA  Leu Upstream Check F: 5` – GAAGTTGGTGACGCGATTGT  Leu Downstream Check F: 5` – TTCCCCTTCAATGTATGCAA |
| *MNN9* | Knockout | Upstream F: 5’ – ATGATTATGGATGGAGTTGATT  Upstream R: 5’ – CACGGCGCGCCTAGCAGCGGATATTCGTTTCCTGTTTTGTTT  Auxotrophic marker F: 5’ – CCGCTGCTAGGCGCGCCGTGACCAGTGTGATGGATATCTGC  Auxotrophic marker R: 5’ – GCAGGGATGCGGCCGCTGACAGCTCGGATCCACTAGTAACG  Downstream F: 5’ – GTCAGCGGCCGCATCCCTGCTCCCATTATACACAGTGAATTT  Downstream R: 5’ – TCAAAATGACCAACATATAAAGA  Nested Fusion F: 5’ – CAAATTGTTGCTACAACTAGGA  Nested Fusion R: 5’ – ATTTGGATAACCACAAATCAAT  Internal Check F: 5’ – TTCGTGGTTTATCTTTTCTTCT  Internal Check R: 5’ – CTCTATGAACATCAGCATTGAC  Upstream Check F: 5’ – TGAAAGTTTGAATGAACAAAAA  Downstream Check R: 5’ – TTGAACATCAACATCAGCTT  His Upstream Check F: 5` – AAAATCAATGGGCATTCTCG  His Downstream Check R: 5` – TGGGAAGCAGACATTCAACA  Leu Upstream Check F: 5` – GAAGTTGGTGACGCGATTGT  Leu Downstream Check F: 5` – TTCCCCTTCAATGTATGCAA |
| *MNN9* | Compliment | Upstream/ORF F: 5’ – TGATTAAGTTTGATATGATGGAA  Upstream/ORF R: 5’ – CACGGCGCGCCTAGCAGCGGACCTAATAGTTATAGAACACAACAATG  Antibiotic marker F: 5’ – CCGCTGCTAGGCGCGCCGTGGATATCAAGCTTGCCTCGTCC  Antibiotic marker R: 5’ – GCAGGGATGCGGCCGCTGACTTACTTTCTGCGCACTTAACTTC  Downstream F: 5’ – GTCAGCGGCCGCATCCCTGCAATATACTTCCCATTATACACAGTGA  Downstream R: 5’ – TATTTATAATCAAAATGACCAACA  Nested Fusion F: 5’ – AATGATGATTATGGATGGAGTT  Nested Fusion R: 5’ –TAAGGTTGAACATCAACATCAG  Internal Check F: 5’ – TTCGTGGTTTATCTTTTCTTCT  Internal Check R: 5’ – CTCTATGAACATCAGCATTGAC  Upstream Check F: 5’ – ATTTATGGAAACTCATGGAGAA  Downstream Check R: 5’ – ATATTCGTTTCCTGTTTTGTTT  Nourseothricin Upstream Check F: 5` – TCGTTGTTGTTTCTTATTCTGG  Nourseothricin Downstream Check R: 5` – TCAGTAAATCCTTCAGCAGTAA |
| *MNN11* | Knockout | Upstream F: 5’ – CAGCATCTAGTGAAAATAGCAA  Upstream R: 5’ – CACGGCGCGCCTAGCAGCGGAGCCAATCTTTGATAAGTTGAT  Auxotrophic marker F: 5’ – CCGCTGCTAGGCGCGCCGTGACCAGTGTGATGGATATCTGC  Auxotrophic marker R: 5’ – GCAGGGATGCGGCCGCTGACAGCTCGGATCCACTAGTAACG  Downstream F: 5’ – GTCAGCGGCCGCATCCCTGCAAAACAAGAACACGAAAATCAT  Downstream R: 5’ – ACTTTATTGCTGATGGAGAAGT  Nested Fusion F: 5’ – AATATCGAGGTCATTTCTTTTG  Nested Fusion R: 5’ – ATTATGAAGTGTTTTCCGAATC  Internal Check F: 5’ – TAACTTATCCAAAAGGTCATCC  Internal Check R: 5’ – GTTTTTACCAACATCCTTGAAT  Upstream Check F: 5’ – AGAGAGGTGGGATTACCCTA  Downstream Check R: 5’ – GGAACCATTACAAAAACTGC  His Upstream Check F: 5` – AAAATCAATGGGCATTCTCG  His Downstream Check R: 5` – TGGGAAGCAGACATTCAACA  Leu Upstream Check F: 5` – GAAGTTGGTGACGCGATTGT  Leu Downstream Check F: 5` – TTCCCCTTCAATGTATGCAA |
| *PHR1* | Knockout | Upstream F: 5’ – TGTAAATAATTGCTGCAAAAAG  Upstream R: 5’ – CACGGCGCGCCTAGCAGCGGTATGTGGATAAGGATTGTAGCA  Auxotrophic marker F: 5’ – CCGCTGCTAGGCGCGCCGTGACCAGTGTGATGGATATCTGC  Auxotrophic marker R: 5’ – GCAGGGATGCGGCCGCTGACAGCTCGGATCCACTAGTAACG  Downstream F: 5’ – GTCAGCGGCCGCATCCCTGCTTGTCTGTTTTCTAAATTCGTG  Downstream R: 5’ – AATATCTTTCAAAAATGGGATG  Nested Fusion F: 5’ – ATTATCCTCATCTACTCGTTGG  Nested Fusion R: 5’ – AAATTGGAAGAAGATGGTATTG  Internal Check F: 5’ – TAGAGAAATCCCAGTTGGTTAC  Internal Check R: 5’ – TGTACCAGAACCAGAACTAACA  Upstream Check F: 5’ – CCCTGTCTTGTTTTTACGAC  Downstream Check R: 5’ – AGGATCCGGATTAGGTTTAG  His Upstream Check F: 5` – AAAATCAATGGGCATTCTCG  His Downstream Check R: 5` – TGGGAAGCAGACATTCAACA  Leu Upstream Check F: 5` – GAAGTTGGTGACGCGATTGT  Leu Downstream Check F: 5` – TTCCCCTTCAATGTATGCAA |
| *PMR1* | Knockout | Upstream F: 5’ – AATTGTGGAGATGGTAAAGAAG  Upstream R: 5’ – CACGGCGCGCCTAGCAGCGGAAGGAGTGTTGTGTGTTGTAAA  Auxotrophic marker F: 5’ – CCGCTGCTAGGCGCGCCGTGACCAGTGTGATGGATATCTGC  Auxotrophic marker R: 5’ – GCAGGGATGCGGCCGCTGACAGCTCGGATCCACTAGTAACG  Downstream F: 5’ – GTCAGCGGCCGCATCCCTGCCCAGAAGTGTGGTATGTGTGTA  Downstream R: 5’ – ATTTCTAGGTTGTGCTAATGGT  Nested Fusion F: 5’ – TTAAAGAGTTGGGTAAAAATGG  Nested Fusion R: 5’ – TTCCTTCTAATAATCCAACACC  Internal Check F: 5’ – AACTTTGGGTTCTGTTAATGTC  Internal Check R: 5’ – AATACCAATATCAGCCAATTTC  Upstream Check F: 5’ – TAATTTTGTTGATGTTGAAGGT  Downstream Check R: 5’ – GGTGCATGATGATAATTTGTAA  His Upstream Check F: 5` – AAAATCAATGGGCATTCTCG  His Downstream Check R: 5` – TGGGAAGCAGACATTCAACA  Leu Upstream Check F: 5` – GAAGTTGGTGACGCGATTGT  Leu Downstream Check F: 5` – TTCCCCTTCAATGTATGCAA |
| *VAN1* | Knockout | Upstream F: 5’ – CAGAACGCGTAAAATATATGAA  Upstream R: 5’ – CACGGCGCGCCTAGCAGCGGATCCTTGGTAGTCATTCAAGTC  Auxotrophic marker F: 5’ – CCGCTGCTAGGCGCGCCGTGACCAGTGTGATGGATATCTGC  Auxotrophic marker R: 5’ – GCAGGGATGCGGCCGCTGACAGCTCGGATCCACTAGTAACG  Downstream F: 5’ – GTCAGCGGCCGCATCCCTGCCACCTCCTTGATTATCTTTGAT  Downstream R: 5’ – AAGTATTTAGTGGCTCATTTGC  Nested Fusion F: 5’ – GAAACGTGATGAAATAATGAAA  Nested Fusion R: 5’ – TATTTGCTGGTGTAATGAGTGT  Internal Check F: 5’ – TCTGACTGTTCTCCAGATGATA  Internal Check R: 5’ – AAATCATCTTCACTAGGCTCAT  Upstream Check F: 5’ – ATATAGGAGTCATTTATCAGGTGAA  Downstream Check R: 5’ – CTATTAGCGCTATCGGTTATTC  His Upstream Check F: 5` – AAAATCAATGGGCATTCTCG  His Downstream Check R: 5` – TGGGAAGCAGACATTCAACA  Leu Upstream Check F: 5` – GAAGTTGGTGACGCGATTGT  Leu Downstream Check F: 5` – TTCCCCTTCAATGTATGCAA |
| *VAN1* | Compliment | Upstream/ORF F: 5’ – TAGGAGTCATTTATCAGGTGAA  Upstream/ORF R: 5’ – CACGGCGCGCCTAGCAGCGGTAATCAAGGAGGTGCTAACTCT  Antibiotic marker F: 5’ – CCGCTGCTAGGCGCGCCGTGGATATCAAGCTTGCCTCGTCC  Antibiotic marker R: 5’ – GCAGGGATGCGGCCGCTGACTTACTTTCTGCGCACTTAACTTC  Downstream F: 5’ – GTCAGCGGCCGCATCCCTGCTTCTGATTTATTGTTTAGTGCAA  Downstream R: 5’ – GAAGAAACAGGAATTTCAAGTAA  Nested Fusion F: 5’ – GCGGGAAAGGAAGACAAAA  Nested Fusion R: 5’ – GACCACTTATATTCTCCTTGGT  Internal Check F: 5’ – TCTGACTGTTCTCCAGATGATA  Internal Check R: 5’ – AAATCATCTTCACTAGGCTCAT  Upstream Check F: 5’ – GCAGAACGCGTAAAATATATGA  Downstream Check R: 5’ – ATCCTTGGTAGTCATTCAAGTC  Nourseothricin Upstream Check F: 5` – CTTTGGATGGTTCTTTCACTAC  Nourseothricin Downstream Check R: 5` – ATTCACATGGATTACAAAACCC |
| *XOG1* | Compliment | Upstream F: 5’ – CGCACAACAGATTAAATTAGAA  Upstream R: 5’ – CACGGCGCGCCTAGCAGCGGAAAGCAATAATAAAACGTGGAT  Auxotrophic marker F: 5’ – CCGCTGCTAGGCGCGCCGTGACCAGTGTGATGGATATCTGC  Auxotrophic marker R: 5’ – GCAGGGATGCGGCCGCTGACAGCTCGGATCCACTAGTAACG  Downstream F: 5’ – GTCAGCGGCCGCATCCCTGCTCGTCGGTTTATAGAGATTTTT  Downstream R: 5’ – GTCTGATGTATTTGAACACGAG  Nested Fusion F: 5’ – TGTTTGTTTTGTTTTGTGGTAT  Nested Fusion R: 5’ – GGTAGTGGAAAATGTTGAAAGT  Internal Check F: 5’ – CAGTTAATTAATGCTGTGGCTA  Internal Check R: 5’ – GTTGATCAATGTCACGAGATAA  Upstream Check F: 5’ – TTTTTCTTCGTCTCCAACAC  Downstream Check R: 5’ – CCTAATGTTGTTTCCTTACCTC  His Upstream Check F: 5` – AAAATCAATGGGCATTCTCG  His Downstream Check R: 5` – TGGGAAGCAGACATTCAACA  Leu Upstream Check F: 5` – GAAGTTGGTGACGCGATTGT  Leu Downstream Check F: 5` – TTCCCCTTCAATGTATGCAA |
|  |  |  |

***C. parapsilosis***

| **Gene** | **Function** | **Primers** |
| --- | --- | --- |
| *ALG11* | Knockout | Upstream F: 5’ – ATATGCAATGTGCCATTTAG  Upstream R: 5’ –CACGGCGCGCCTAGCAGCGGTGGACGAGTGCTCTCTCTTA  Auxotrophic marker F: 5’ – CCGCTGCTAGGCGCGCCGTGACCAGTGTGATGGATATCTGC  Auxotrophic marker R: 5’ – GCAGGGATGCGGCCGCTGACAGCTCGGATCCACTAGTAACG  Downstream F: 5’ – GTCAGCGGCCGCATCCCTGCTTGCAATCGTTTGTCTGTAT  Downstream R: 5’ – TGGGTAACTTTTTCACCAGT  Nested Fusion F: 5’ – CAAAATGTTGTTGTAAACACG  Nested Fusion R: 5’ – ACACGAATTTGGAAATCATC  Internal Check F: 5’ – CTAGTCCCCTCACAGAAATG  Internal Check R: 5’ – AGTGGAAAGTAGTTGCTCCA  Upstream Check F: 5’ – ATTGCGTATCGTGGATATAG  Downstream Check R: 5’ – GAAAATTGCAAACGATACAT  His Upstream Check F: 5` – AAAATCAATGGGCATTCTCG  His Downstream Check R: 5` – TGGGAAGCAGACATTCAACA  Leu Upstream Check F: 5` – GAAGTTGGTGACGCGATTGT  Leu Downstream Check F: 5` – TTCCCCTTCAATGTATGCAA |
| *BGL2* | Knockout | Upstream F: 5’ – AATGATTGTATCGCGAAAGT  Upstream R: 5’ –CACGGCGCGCCTAGCAGCGGTTGCAAGATTTTGTTGTTGA  Auxotrophic marker F: 5’ – CCGCTGCTAGGCGCGCCGTGACCAGTGTGATGGATATCTGC  Auxotrophic marker R: 5’ – GCAGGGATGCGGCCGCTGACAGCTCGGATCCACTAGTAACG  Downstream F: 5’ – GTCAGCGGCCGCATCCCTGCACTGGTGAGTTTTCATTCGT  Downstream R: 5’ – GATTTCAACCACATCCAAAT  Nested Fusion F: 5’ – ATGCTAGGGGAAATTTAAGG  Nested Fusion R: 5’ – AACACCACTTTCCGACATAC  Internal Check F: 5’ – TATCCAACCAAACAACAACA  Internal Check R: 5’ – CAATGCTTTTCAACATCTGA  Upstream Check F: 5’ – CCTTCAAGAGCAAATGTATC  Downstream Check R: 5’ – CCACAACAGCACTTCTTATT  His Upstream Check F: 5` – AAAATCAATGGGCATTCTCG  His Downstream Check R: 5` – TGGGAAGCAGACATTCAACA  Leu Upstream Check F: 5` – GAAGTTGGTGACGCGATTGT  Leu Downstream Check F: 5` – TTCCCCTTCAATGTATGCAA |
| *MNN4-4* | Knockout | Upstream F: 5’ – TTTGGTTGTTACTGGAGACC  Upstream R: 5’ –CACGGCGCGCCTAGCAGCGGGTGACTTGCTTGTGTCAAAC  Auxotrophic marker F: 5’ – CCGCTGCTAGGCGCGCCGTGACCAGTGTGATGGATATCTGC  Auxotrophic marker R: 5’ – GCAGGGATGCGGCCGCTGACAGCTCGGATCCACTAGTAACG  Downstream F: 5’ – GTCAGCGGCCGCATCCCTGCTAGGGTTGGTTACTCAAGCA  Downstream R: 5’ – TGCCACGTTTCAACATAAT  Nested Fusion F: 5’ – CATCTATATCCACTCCTCGAA  Nested Fusion R: 5’ – ACAACTGATGGGTTTTCAAC  Internal Check F: 5’ – GAAAAAGTTTTGTTGGATCG  Internal Check R: 5’ – ATCTCAGTTTGCGTTTTCAT  Upstream Check F: 5’ – GGGGATCTTCACTAACAAAT  Downstream Check R: 5’ – AGGCGGTAGAAATTCAAG  His Upstream Check F: 5` – AAAATCAATGGGCATTCTCG  His Downstream Check R: 5` – TGGGAAGCAGACATTCAACA  Leu Upstream Check F: 5` – GAAGTTGGTGACGCGATTGT  Leu Downstream Check F: 5` – TTCCCCTTCAATGTATGCAA |
| *MNN9* | Compliment | Upstream F: 5’ – AAGGAAAAGAAGGAGAAAGC  Upstream R: 5’ –CACGGCGCGCCTAGCAGCGGGGGATATATCAACGCAATGT  Auxotrophic marker F: 5’ – CCGCTGCTAGGCGCGCCGTGACCAGTGTGATGGATATCTGC  Auxotrophic marker R: 5’ – GCAGGGATGCGGCCGCTGACAGCTCGGATCCACTAGTAACG  Downstream F: 5’ – GTCAGCGGCCGCATCCCTGCAACGGTAACACAGGTGTAGG  Downstream R: 5’ – GCGTCTTTGTTTTCCTTAGT  Nested Fusion F: 5’ – TTGGATGAGTGGAAGAGAAC  Nested Fusion R: 5’ – CAGCTTAGAATCGCTTGACT  Internal Check F: 5’ – CCAAGAAGGAAGAAGTGTTG  Internal Check R: 5’ – TCAAGTGGTAAAATGGGAAC  Upstream Check F: 5’ – CTAAAAAGGAAGCTTCTGC  Downstream Check R: 5’ – TGATTTCCTGATTTGTTGTC  His Upstream Check F: 5` – AAAATCAATGGGCATTCTCG  His Downstream Check R: 5` – TGGGAAGCAGACATTCAACA  Leu Upstream Check F: 5` – GAAGTTGGTGACGCGATTGT  Leu Downstream Check F: 5` – TTCCCCTTCAATGTATGCAA |
| *MNN11* | Knockout | Upstream F: 5’ – GTGATCAAATTCGTCTTGGT  Upstream R: 5’ –CACGGCGCGCCTAGCAGCGGATTTGGATGATGGTTGATGT  Auxotrophic marker F: 5’ – CCGCTGCTAGGCGCGCCGTGACCAGTGTGATGGATATCTGC  Auxotrophic marker R: 5’ – GCAGGGATGCGGCCGCTGACAGCTCGGATCCACTAGTAACG  Downstream F: 5’ – GTCAGCGGCCGCATCCCTGCTGTATCTAAAGACAAGCTTTGAA  Downstream R: 5’ – CTACAACTTTCCCAAACAGG  Nested Fusion F: 5’ – TGCAAAGAATATGAACACTCC  Nested Fusion R: 5’ – ACACTTGACGGTTCAAGAAG  Internal Check F: 5’ – AGTTGAACAAGCACCACTTT  Internal Check R: 5’ – CTTCACAATCGGAAATCAGT  Upstream Check F: 5’ – TGATTACTGTTTTGGTTGCT  Downstream Check R: 5’ – ATGTGAGACTGTTGCTCAAT  His Upstream Check F: 5` – AAAATCAATGGGCATTCTCG  His Downstream Check R: 5` – TGGGAAGCAGACATTCAACA  Leu Upstream Check F: 5` – GAAGTTGGTGACGCGATTGT  Leu Downstream Check F: 5` – TTCCCCTTCAATGTATGCAA |
| *PHR1* | Knockout | Upstream F: 5’ – TTTCTCCTAACAAGGTCACG  Upstream R: 5’ –CACGGCGCGCCTAGCAGCGGTAGTGGTAGTGCGAAGTGTG  Auxotrophic marker F: 5’ – CCGCTGCTAGGCGCGCCGTGACCAGTGTGATGGATATCTGC  Auxotrophic marker R: 5’ – GCAGGGATGCGGCCGCTGACAGCTCGGATCCACTAGTAACG  Downstream F: 5’ – GTCAGCGGCCGCATCCCTGCAAATGAAAGAAAAAGGCTGA  Downstream R: 5’ – AGACAGACAATGCAGGACTT  Nested Fusion F: 5’ – ATAGTCGTCTCGCCACTTTA  Nested Fusion R: 5’ – ACAAGACGTTGGATGAATGT  Internal Check F: 5’ – TGGACCATGATGAATGTATG  Internal Check R: 5’ – GATGGGAGCAGAATGAGTAG  Upstream Check F: 5’ – GTGGTAGACATGAATTGCTC  Downstream Check R: 5’ – TTGGACACCAGTATTTATCC  His Upstream Check F: 5` – AAAATCAATGGGCATTCTCG  His Downstream Check R: 5` – TGGGAAGCAGACATTCAACA  Leu Upstream Check F: 5` – GAAGTTGGTGACGCGATTGT  Leu Downstream Check F: 5` – TTCCCCTTCAATGTATGCAA |
| *PMR1* | Knockout | Upstream F: 5’ – CCCAATGCAAAGGTTATACT  Upstream R: 5’ –CACGGCGCGCCTAGCAGCGGTATATCTTTCGGGTGTTTGG  Auxotrophic marker F: 5’ – CCGCTGCTAGGCGCGCCGTGACCAGTGTGATGGATATCTGC  Auxotrophic marker R: 5’ – GCAGGGATGCGGCCGCTGACAGCTCGGATCCACTAGTAACG  Downstream F: 5’ – GTCAGCGGCCGCATCCCTGCATCGAATGCAAACTCTTGTT  Downstream R: 5’ – TACGAGGAGGAAAACTCAAG  Nested Fusion F: 5’ – ATGAGGAGGATAAGGTGGAT  Nested Fusion R: 5’ – TAAGCAATTCCCCATTACAC  Internal Check F: 5’ – GCAAAGAGAGAGGGTGTATG  Internal Check R: 5’ – AATCCGAAAAACGTACTCAA  Upstream Check F: 5’ – ATGTTGAAGGCATTAAAGTG  Downstream Check R: 5’ – CATTTTAGAAATGCCAAACA  His Upstream Check F: 5` – AAAATCAATGGGCATTCTCG  His Downstream Check R: 5` – TGGGAAGCAGACATTCAACA  Leu Upstream Check F: 5` – GAAGTTGGTGACGCGATTGT  Leu Downstream Check F: 5` – TTCCCCTTCAATGTATGCAA |
| *VAN1* | Knockout | Upstream F: 5’ – GACAAGCTAGAGCAAGGGTA  Upstream R: 5’ –CACGGCGCGCCTAGCAGCGGAGGTAATGACGTTGACGTTT  Auxotrophic marker F: 5’ – CCGCTGCTAGGCGCGCCGTGACCAGTGTGATGGATATCTGC  Auxotrophic marker R: 5’ – GCAGGGATGCGGCCGCTGACAGCTCGGATCCACTAGTAACG  Downstream F: 5’ – GTCAGCGGCCGCATCCCTGCATCAGTAGGAAGGCAGGATT  Downstream R: 5’ – TTTGAACCTGCTGTTTTCTT  Nested Fusion F: 5’ – ACCCCCAAGATATTTAAAGC  Nested Fusion R: 5’ – GTGTATTTGGGCTATGCAAT  Internal Check F: 5’ – ACAACCGAAGATTTGAAAAA  Internal Check R: 5’ – CACTTCACGAGGATTACCAT  Upstream Check F: 5’ – TGGTTTAAAGACTTGAATGG  Downstream Check R: 5’ – GTTTTCTTGGGTGGTTTTAT  His Upstream Check F: 5` – AAAATCAATGGGCATTCTCG  His Downstream Check R: 5` – TGGGAAGCAGACATTCAACA  Leu Upstream Check F: 5` – GAAGTTGGTGACGCGATTGT  Leu Downstream Check F: 5` – TTCCCCTTCAATGTATGCAA |
| *XOG1* | Knockout | Upstream F: 5’ – TGGCATACAAAAGAGAACAA  Upstream R: 5’ –CACGGCGCGCCTAGCAGCGGATGGTTGTTGTAAATGTGACC  Auxotrophic marker F: 5’ – CCGCTGCTAGGCGCGCCGTGACCAGTGTGATGGATATCTGC  Auxotrophic marker R: 5’ – GCAGGGATGCGGCCGCTGACAGCTCGGATCCACTAGTAACG  Downstream F: 5’ – GTCAGCGGCCGCATCCCTGCTTGGTTAATTTTGGTTTGGT  Downstream R: 5’ – ACGTGACTGTGATGTTTGAA  Nested Fusion F: 5’ – AACTCATGACACATCCACAA  Nested Fusion R: 5’ – TCTTGTGCAAACACCGTAT  Internal Check F: 5’ – ACAAAGCAATTGGGTAAAGA  Internal Check R: 5’ – GAAATAAACCAGCAGCAGTC  Upstream Check F: 5’ – TGTGTGTGTGCTAAGCTAAA  Downstream Check R: 5’ – TGAATTTCTCGATCTTTTTG  His Upstream Check F: 5` – AAAATCAATGGGCATTCTCG  His Downstream Check R: 5` – TGGGAAGCAGACATTCAACA  Leu Upstream Check F: 5` – GAAGTTGGTGACGCGATTGT  Leu Downstream Check F: 5` – TTCCCCTTCAATGTATGCAA |
|  |  |  |

***C. glabrata***

| **Gene** | **Function** | **Primers** |
| --- | --- | --- |
| *ALG11* | Knockout | Upstream F: 5’ – TCTCCTCTTTGGATGGTAGA  Upstream R: 5’ – CCGCTGCTAGGCGCGCCGTGTGTTGCTAAACGCTAACTCA  Antibiotic marker F: 5’ – CACGGCGCGCCTAGCAGCGGCAGAATACCCTCCTTGACAG  Antibiotic marker R: 5’ – GTCAGCGGCCGCATCCCTGCGCGGCGTTAGTATCGAAT  Downstream F: 5’ – GCAGGGATGCGGCCGCTGACCATGTGTTGCCATCATCTAC  Downstream R: 5’ – GCCTACAGTTAAGGAATCTGG  Nested Fusion F: 5’ – ATATCTGTTGGACCCATTGT  Nested Fusion R: 5’ – GGTTTTCCTTCTTCTTACCTG  Internal Check F: 5’ – GGTGAGAAAGTTCTGTGGAA  Internal Check R: 5’ – ATATCTGCCAGAGAGCCATA  Upstream Check F: 5’ – AAGGCGACTTTACCAAAC  Downstream Check R: 5’ – ATGTACCGAAAGATCAGAC  Nourseothricin Upstream Check F: 5` - CAACTGGAACTTCTCTCAAA  Nourseothricin Downstream Check R: 5` - CATTTGTGGTTGGAAGTTAC |
| *BGL2* | Knockout | Upstream F: 5’ – TCATCACTTCTCACCACAAA  Upstream R: 5’ – CCGCTGCTAGGCGCGCCGTGAGAGTACCAAGGTTAGTGTGCT  Antibiotic marker F: 5’ – CACGGCGCGCCTAGCAGCGGCAGAATACCCTCCTTGACAG  Antibiotic marker R: 5’ – GTCAGCGGCCGCATCCCTGCGCGGCGTTAGTATCGAAT  Downstream F: 5’ – GCAGGGATGCGGCCGCTGACTCGAATTCGGTTATGACTTT  Downstream R: 5’ – AGACTGATGGAAAAATCCAA  Nested Fusion F: 5’ – AGGAGGCACAGAGAAACAC  Nested Fusion R: 5’ – TAGTTTTGGAGAAGGGTGAG  Internal Check F: 5’ – CTGTTGCAGCTTTAGCATTT  Internal Check R: 5’ – CAGTGCTTTTCAACATCAGA  Upstream Check F: 5’ – TTCGTCAAGAGACTAGATGG  Downstream Check R: 5’ – ACAGGTTGAAAACCATTTTA  Nourseothricin Upstream Check F: 5` - CAACTGGAACTTCTCTCAAA  Nourseothricin Downstream Check R: 5` - CATTTGTGGTTGGAAGTTAC |
| *BIG1* | Knockout | Upstream F: 5’ – ATCTTGCCAGGTCTCAGTAA  Upstream R: 5’ – CCGCTGCTAGGCGCGCCGTGTCTTGACTGCCATTAAACAG  Antibiotic marker F: 5’ – CACGGCGCGCCTAGCAGCGGCAGAATACCCTCCTTGACAG  Antibiotic marker R: 5’ – GTCAGCGGCCGCATCCCTGCGCGGCGTTAGTATCGAAT  Downstream F: 5’ – GCAGGGATGCGGCCGCTGACCAGTTGAAGTATGATCTTGCATA  Downstream R: 5’ – TCAACTGGATAGAAGGTCGT  Nested Fusion F: 5’ – CTGCTTCAAAGAGGCTAGAA  Nested Fusion R: 5’ – AGTGATGTTTCAGCTCTGGT  Internal Check F: 5’ – GCTCTAGCGAGTAGTCTGGA  Internal Check R: 5’ – CGTAGTATTTTTGGCGCTAT  Upstream Check F: 5’ – ACCTGCACAGTATTCCATT  Downstream Check R: 5’ – CTGGTGACGTTGTAAGAAAG  Nourseothricin Upstream Check F: 5` - CAACTGGAACTTCTCTCAAA  Nourseothricin Downstream Check R: 5` - CATTTGTGGTTGGAAGTTAC |
| *BIG1* | Compliment | Upstream/ORF F: 5’ – TTCATCTATCTTGCCAGGTCTC  Upstream/ORF R: 5’ – CACGGCGCGCCTAGCAGCGGGAGAGCTAGTTTCCCCTCT  Antibiotic marker F: 5’ – CCGCTGCTAGGCGCGCCGTGGTATAGTGCTTGCTGTTCGAT  Antibiotic marker R: 5’ – GCAGGGATGCGGCCGCTGACATTTTATGATGGAATGAATGG  Downstream F: 5’ – GTCAGCGGCCGCATCCCTGCATTACAGTTGAAGTATGATCTTG  Downstream R: 5’ – GCTGGCATGATATGGAGATT  Nested Fusion F: 5’ – TATTCGGTATCTGCTTCAAAGA  Nested Fusion R: 5’ – GTGGTAAGAGTATTGTAGCTGA  Internal Check F: 5’ – GCTCTAGCGAGTAGTCTGGA  Internal Check R: 5’ – CGTAGTATTTTTGGCGCTAT  Upstream Check F: 5’ – ACCTGCACAGTATTCCATTATA  Downstream Check R: 5’ – AATAATGCGTGAATTTGTGACT  HygB Upstream Check F: 5` - TGGAAATCTGGAAATCTGGTT  HygB Downstream Check R: 5` - ATCGGTATCAATGCCTTCTATC |
| *MNN4-4* | Knockout | Upstream F: 5’ – TCATTATGAACCTGCAGTGA  Upstream R: 5’ – CCGCTGCTAGGCGCGCCGTGAGCATTCAAATCCCTTCTTA  Antibiotic marker F: 5’ – CACGGCGCGCCTAGCAGCGGCAGAATACCCTCCTTGACAG  Antibiotic marker R: 5’ – GTCAGCGGCCGCATCCCTGCGCGGCGTTAGTATCGAAT  Downstream F: 5’ – GCAGGGATGCGGCCGCTGACTATTTTAAAGGGCATGTTGG  Downstream R: 5’ – TATGAAACCGATGAACAATG  Nested Fusion F: 5’ – AGACATTATTTTGGTGTTGC  Nested Fusion R: 5’ – GTTATCACCCGTTGACATAG  Internal Check F: 5’ – AATCTCGAGTCAACTGTGCT  Internal Check R: 5’ – TTTCCTTCCCTAGGATCTTC  Upstream Check F: 5’ – CTCTACTCAGACACCGAAGA  Downstream Check R: 5’ – CAGTTCAAGATGAAGTTTCG  Nourseothricin Upstream Check F: 5` - CAACTGGAACTTCTCTCAAA  Nourseothricin Downstream Check R: 5` - CATTTGTGGTTGGAAGTTAC |
| *MNN9* | Knockout | Upstream F: 5’ – AAAGAGACGAGGACAAGGTT  Upstream R: 5’ – CCGCTGCTAGGCGCGCCGTGAAGAGGCTCCTTTCTTGTTT  Antibiotic marker F: 5’ – CACGGCGCGCCTAGCAGCGGCAGAATACCCTCCTTGACAG  Antibiotic marker R: 5’ – GTCAGCGGCCGCATCCCTGCGCGGCGTTAGTATCGAAT  Downstream F: 5’ – GCAGGGATGCGGCCGCTGACATGTGGTTGCCCTAATGTAA  Downstream R: 5’ – AAGACAACTTTCGGACTTGA  Nested Fusion F: 5’ – GTATCAGAAGCAGAAGGTGA  Nested Fusion R: 5’ – AATAACCGCAGCATACTAAA  Internal Check F: 5’ – CTGATTTTCAGGTCGGATAG  Internal Check R: 5’ – GAAAGTTGGGAACATAGCAC  Upstream Check F: 5’ – CAGAAGCAGAAGCAGTAGC  Downstream Check R: 5’ – CGACTTTTCTCGAGACACTA  Nourseothricin Upstream Check F: 5` - CAACTGGAACTTCTCTCAAA  Nourseothricin Downstream Check R: 5` - CATTTGTGGTTGGAAGTTAC |
| *MNN11* | Knockout | Upstream F: 5’ – TGCTCCCTGAACTTTCTAAC  Upstream R: 5’ – CCGCTGCTAGGCGCGCCGTGTTCCAAACAGGTATCAGAAAA  Antibiotic marker F: 5’ – CACGGCGCGCCTAGCAGCGGCAGAATACCCTCCTTGACAG  Antibiotic marker R: 5’ – GTCAGCGGCCGCATCCCTGCGCGGCGTTAGTATCGAAT  Downstream F: 5’ – GCAGGGATGCGGCCGCTGACTTTGGGATTCATTGAGCTAT  Downstream R: 5’ – CACTTACATCGCTTGTCTCA  Nested Fusion F: 5’ – ACAACTGACGTATGGTCATC  Nested Fusion R: 5’ – CACCACTGTTATAAAGAATCAA  Internal Check F: 5’ – GGTACGTGTCGAAGAAGAAG  Internal Check R: 5’ – TTGATGTTCAAGTTTGTTGG  Upstream Check F: 5’ – GTGTACTTGTCCTGCAACTC  Downstream Check R: 5’ – ACTTGGAGACCACCAGTAAT  Nourseothricin Upstream Check F: 5` - CAACTGGAACTTCTCTCAAA  Nourseothricin Downstream Check R: 5` - CATTTGTGGTTGGAAGTTAC |
| *PHR1* | Knockout | Upstream F: 5’ – TTCCCTTCATTGACTATTGG  Upstream R: 5’ – CCGCTGCTAGGCGCGCCGTGCTCCAGGAAAGCAAAAGAT  Antibiotic marker F: 5’ – CACGGCGCGCCTAGCAGCGGCAGAATACCCTCCTTGACAG  Antibiotic marker R: 5’ – GTCAGCGGCCGCATCCCTGCGCGGCGTTAGTATCGAAT  Downstream F: 5’ – GCAGGGATGCGGCCGCTGACAATGGGATCTTGATTATCCTT  Downstream R: 5’ – ACATGGTTCAATATGGAGGA  Nested Fusion F: 5’ – AAGAAAGCAATCTCGACGTA  Nested Fusion R: 5’ – ATATGCCCCAACAACATATC  Internal Check F: 5’ – AGCTTGCCACAAATGTTATT  Internal Check R: 5’ – CGAAGTTCAGCTTTTCCTTA  Upstream Check F: 5’ – CTTAATTAGAGGGAGGAGGA  Downstream Check R: 5’ – GAATACCTAGTATCGCCAGA  Nourseothricin Upstream Check F: 5` - CAACTGGAACTTCTCTCAAA  Nourseothricin Downstream Check R: 5` - CATTTGTGGTTGGAAGTTAC |
| *PMR1* | Knockout | Upstream F: 5’ – AGTTCGAAGACGCAAAGAA  Upstream R: 5’ – CCGCTGCTAGGCGCGCCGTGGTAATAAGCCTTGATTCAGCA  Antibiotic marker F: 5’ – CACGGCGCGCCTAGCAGCGGCAGAATACCCTCCTTGACAG  Antibiotic marker R: 5’ – GTCAGCGGCCGCATCCCTGCGCGGCGTTAGTATCGAAT  Downstream F: 5’ – GCAGGGATGCGGCCGCTGACATTTTGTCGATGAAGCACTC  Downstream R: 5’ – AAACCTGTTTGCTGTGGTAG  Nested Fusion F: 5’ – GACGACTCGCAGATATGG  Nested Fusion R: 5’ – GCATCTGTGGTTGTAAAGGT  Internal Check F: 5’ – CGCTTATATGGGTACTCTGG  Internal Check R: 5’ – TCCACCTCTATGCAGTTTTT  Upstream Check F: 5’ – AGGAGAAGACAGAGGAGAAG  Downstream Check R: 5’ – ATTCAGTTGATTTGGTTGAA  Nourseothricin Upstream Check F: 5` - CAACTGGAACTTCTCTCAAA  Nourseothricin Downstream Check R: 5` - CATTTGTGGTTGGAAGTTAC |
| *VAN1* | Knockout | Upstream F: 5’ – CGAGAGCGATAGTGATAAGG  Upstream R: 5’ – CCGCTGCTAGGCGCGCCGTGCACAACGTAATCGTGTATGC  Antibiotic marker F: 5’ – CACGGCGCGCCTAGCAGCGGCAGAATACCCTCCTTGACAG  Antibiotic marker R: 5’ – GTCAGCGGCCGCATCCCTGCGCGGCGTTAGTATCGAAT  Downstream F: 5’ – GCAGGGATGCGGCCGCTGACTACCGTTTGAATTCGTTTCT  Downstream R: 5’ – GGGTTCGTTTGGTATTTTT  Nested Fusion F: 5’ – AACACCAATCCACTGTAAAA  Nested Fusion R: 5’ – AGACACTATACCCCGCTCT  Internal Check F: 5’ – GCCAAGAAGAAAAACTTCAA  Internal Check R: 5’ – CATCCAAGAATTCAGGTCAT  Upstream Check F: 5’ – GTAGCAATTTCCATATTTCG  Downstream Check R: 5’ – ACATTCCAATTGAATTTAAGAC  Nourseothricin Upstream Check F: 5` - CAACTGGAACTTCTCTCAAA  Nourseothricin Downstream Check R: 5` - CATTTGTGGTTGGAAGTTAC |
| *XOG1* | Knockout | Upstream F: 5’ – CATCTTCGAATCGTTGTTTT  Upstream R: 5’ – CCGCTGCTAGGCGCGCCGTGTCTCCTTGGGTTCTTTAATTT  Antibiotic marker F: 5’ – CACGGCGCGCCTAGCAGCGGCAGAATACCCTCCTTGACAG  Antibiotic marker R: 5’ – GTCAGCGGCCGCATCCCTGCGCGGCGTTAGTATCGAAT  Downstream F: 5’ – GCAGGGATGCGGCCGCTGACAAAGCTGGACAACATTGATT  Downstream R: 5’ – GTCGTCAAATTGTCATTCG  Nested Fusion F: 5’ – CTTCCCTTTCGATATTCCTT  Nested Fusion R: 5’ – TTGGTCTATCAATGCTCAAGT  Internal Check F: 5’ – GTTCAGAACCAACCCTTACA  Internal Check R: 5’ – AGATCCAGCCACCTCTTAAT  Upstream Check F: 5’ – CCGCAAGTAGTCATCTATGT  Downstream Check R: 5’ – TCTTCAACTTCTGAATCGTC  Nourseothricin Upstream Check F: 5` - CAACTGGAACTTCTCTCAAA  Nourseothricin Downstream Check R: 5` - CATTTGTGGTTGGAAGTTAC |
|  |  |  |
